# Next-day manufacture of a novel anti-CD19 CAR-T therapy for B-cell acute lymphoblastic leukemia: first-in-human clinical study

**DOI:** 10.1038/s41408-022-00694-6

**Published:** 2022-07-07

**Authors:** Junfang Yang, Jiaping He, Xian Zhang, Jingjing Li, Zhenguang Wang, Yongliang Zhang, Liyuan Qiu, Qionglu Wu, Zhe Sun, Xun Ye, Wenjie Yin, Wei Cao, Lianjun Shen, Martina Sersch, Peihua Lu

**Affiliations:** 1 Hebei Yanda Lu Daopei Hospital, Langfang, Hebei China; 2 Beijing Lu Daopei Institute of Hematology, Beijing, China; 3 Gracell Biotechnologies Co., Ltd, Shanghai, China

**Keywords:** Immunotherapy, Phase I trials

## Abstract

To improve clinical outcomes and shorten the vein-to-vein time of chimeric antigen receptor T (CAR-T) cells, we developed the FasT CAR-T (F-CAR-T) next-day manufacturing platform. We report the preclinical and first-in-human clinical studies evaluating the safety, feasibility, and preliminary efficacy of CD19 F-CAR-T in B-cell acute lymphoblastic leukemia (B-ALL). CD19 F-CAR-T cells demonstrated excellent proliferation with a younger cellular phenotype, less exhaustion, and more effective tumor elimination compared to conventional CAR-T cells in the preclinical study. In our phase I study (NCT03825718), F-CAR-T cells were successfully manufactured and infused in all of the 25 enrolled pediatric and adult patients with B-ALL. CD19 F-CAR-T safety profile was manageable with 24% grade 3 cytokine release syndrome (CRS) and 28% grade 3/4 neurotoxicity occurring predominantly in pediatric patients. On day 14, 23/25 patients achieved minimal residual disease (MRD)-negative complete remission (CR), and 20 subsequently underwent allogeneic hematopoietic stem cell transplantation (allo-HSCT) within 3 months post F-CAR-T therapy. Fifteen of 20 patients were disease-free with a median remission duration of 734 days. One patient relapsed and 4/20 died from transplant-related mortality. Of the three patients who did not undergo allo-HSCT, two remained in CR until 10 months post-F-CAR-T. Our data indicate that anti-CD19 FasT CAR-T shows promising early efficacy for B-ALL. Further evaluations in larger clinical studies are needed.

## Introduction

CD19-targeting chimeric antigen receptor-engineered T cells (CAR-T) represent a major advancement in refractory/relapsed (R/R) B-cell acute lymphocytic leukemia (B-ALL) with high initial complete remission (CR) rate of around 70–90% [1–8]. However, current CAR-T cell manufacturing requires a long waiting time for patients, typically requiring a minimum of 7–14 days of manufacturing time [9–12]. Two recent large-scale CD19-targeted CAR-T clinical trials [9, 11] reported that 20–30% of enrolled patients with B-ALL ultimately were not infused with CAR-T cells due to death from rapid disease progression or CAR-T cell manufacturing failure. Thus, shortening the duration between apheresis and CAR-T infusion is critical for patients with R/R B-ALL. Furthermore, the high cost of commercially available CAR-T cell products creates a major access barrier and limits its broad application for patients who could benefit from this novel therapeutic technology.

Notably, about 28–43% of B-ALL patients who achieve CR after CAR-T cell treatment relapse, highlighting the importance of optimizing the duration of remission after CAR-T treatment. Prolonged ex vivo culture and expansion of T- cells are associated with reduced lifespan and potency of CAR-T after adoptive transfer [13–17]. A recent study [18] reported that CAR-T cells manufactured within 3 days exerted enhanced proliferative capacity and increased anti-leukemia as compared to those produced within 5 or 9 days in a preclinical study. However, the feasibility of accelerated CAR-T production and its efficacy and potency in the clinic have not been widely tested [9–11].

To shorten the manufacturing time, minimize the cost, and optimize the function of CAR-T cell therapy, the novel anti-CD19 CAR-T therapy called FasT CAR-T (F-CAR-T) was developed with a significantly expedited CAR-T next-day manufacturing process. Here, we describe the preclinical and phase I clinical study of CD19 F-CAR-T therapy in R/R B-ALL patients.

## Methods

### F-CAR-T cell manufacturing

Peripheral blood mononuclear cells (PBMCs) were obtained from healthy donors and enrolled patients by leukapheresis using COM.TEC (Fresenius Kabi, USA) or COBE Spectra (Terumo BCT, USA). Within 30 h after harvest, PBMCs were transported to the GMP-compliant facility at Gracell Biotechnologies, Ltd. in Shanghai under 18–25 °C conditions. T-cells from PBMCs were isolated using Dynabeads CD3/CD28 CTS (Thermo Fisher Scientific, USA), and transduced next day with CD19+ CAR (Fig. S1A) lentiviral vectors in X-vivo culture medium containing IL-2. CAR-T cells were collected the next day without an expansion step and washed with saline. F-CAR-T products and quality control (QC) samples were harvested and cryopreserved in a cell freezing medium in Forma CryoMed Controlled Rate Freezer (Thermo Scientific). CAR expression and positive rate were determined after in vitro culture for 3 days. After passing all tests required for release, frozen F-CAR-T products were transported to the hospital for infusion. The release criteria are summarized in Table S1. In short, the manufacturing time for F-CAR-T was the next day plus approximately 7 more days of QC tests and additional time for cell release and transportation (Fig. S1B). Conventional CAR-T (C-CAR-T) cells for animal studies were produced following a published protocol 19]. Details regarding the preclinical studies are provided in Supplementary Methods.

### Study design and participants

We conducted a single-arm, single-center, proof-of-concept phase I clinical trial of CD19 F-CAR-T in 25 pediatric and adult patients with CD19+ R/R B-ALL (https://clinicaltrials.gov, NCT03825718). The primary objective of the study was to assess the safety and feasibility of the F-CAR-T therapy. Secondary objectives included preliminary efficacy. The study was approved by the Institutional Ethics Committee of Hebei Yanda Lu Daopei Hospital and conducted in accordance with the Declaration of Helsinki. Signed informed consent was obtained from each participating patient.

CD19+ B-ALL patients between 2 and 70 years of age with an Eastern Cooperative Oncology Group (ECOG) score of 0–3 were eligible according to the inclusion and exclusion criteria. Patients who relapsed after allogeneic hematopoietic stem cell transplantation (allo-HSCT) without active graft-versus-host disease (GVHD) were also eligible. Patients who previously received CAR-T cell therapy were excluded.

A lymphodepleting chemotherapy regimen of intravenous fludarabine (30 mg/m^2^/d) and cyclophosphamide (250–300 mg/m^2^/d) (FC) for 3 consecutive days (day -5~day -3) was planned prior to CD19 F-CAR-T cell infusion. Bridging chemotherapy to control rapid disease was allowed per protocol. Based on accumulating evidence from published literature [20–22], consolidative allo-HSCT after CD19 CAR-T therapy for B-ALL within 3 months can improve leukemia-free survival (LFS). In the present study, after patients achieved CR with F-CAR-T therapy, consolidative allo-HSCT was allowed. Assessment of F-CAR-T cells response is detailed in the clinical section of Supplementary Methods.

### Assessment and management of adverse effects

Cytokine release syndrome (CRS) and immune effector cell-associated neurotoxicity syndrome (ICANS) were graded according to the ASTCT consensus guideline [23]. Individual organ toxicities were graded in accordance with the National Cancer Institute Common Terminology Criteria for Adverse Events (CTCAE). Version 5.0 [24]. CRS was managed aggressively with corticosteroids, either dexamethasone or methylprednisolone, or both, +/− tocilizumab, if the patient developed CRS with a persisting fever of >39 °C for 24 h or ≥grade 2 CRS regardless of concurrent ≥grade 2 ICANS. Other side effects were managed with standard of care.

### Statistics

Statistical analyses were conducted using GraphPad Prism software 7.0. Unpaired Student’s *t*-test was applied for 2-group comparisons. The Mann–Whitney test was used to compare the mean peak expansion levels of circulating F-CAR-T cells by qPCR. *P*-values < 0.05 were considered statistically significant. For plasma cytokines, fold change between maximum expression level and the baseline expression level was transformed with log10, and then a heatmap was plotted together with corresponding maximum CRS characterization, using the Bioconductor pheatmap package and R language.

## Results

### Preclinical evaluation of FasT CAR-T cells

#### FasT CAR-T (F-CAR-T) proliferation in vitro

**Fig. 1:**
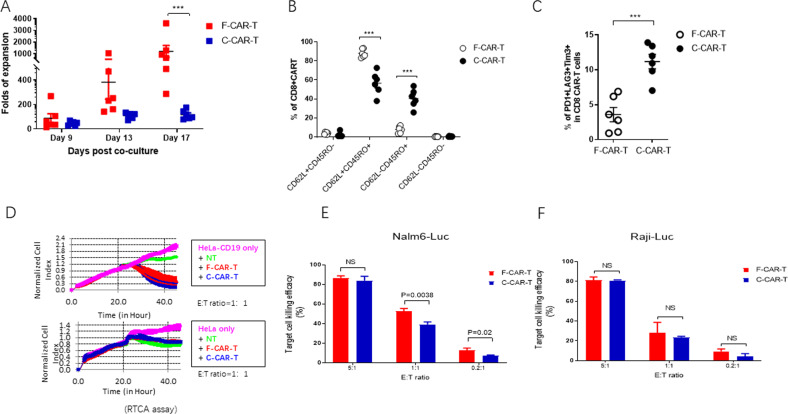
Characterization of F-CAR-T in vitro. **A** Ex vivo cell proliferation of F-CAR-T and C-CAR-T derived from B-ALL patients (*n* = 6) (***P = 0.001, F-CAR-T vs. C-CAR-T, d17, unpaired student two-tailed *t*-test). **B** Tscm, Tcm, and Tem were characterized by surface staining of CD45RO and CD62L and analyzed with flow cytometry (***P < 0.001 comparing F-CAR-T and C-CAR-T). **C** T-cell exhaustion was characterized by PD-1, LAG3, and TIM-3 staining; Statistical analyses of the percentage of PD1^+^ LAG3^+^ Tim3^+^ (***P < 0.001, comparing F-CAR-T and C-CAR-T), unpaired student two-tailed *t*-test). **D** RTCA assay was used to examine the specific killing of HeLa-CD19 cells. Growth of target HeLa-CD19 or HeLa cells were monitored dynamically. **E** CD19+ target Nalm6-Luc cells or **F** Raji-Luc cells were co-cultured with either F-CAR-T or C-CAR-T for 6 h. Target cell killing efficacy was calculated by luciferase activity. NS, *P* > 0.05 F-CAR-T vs. C-CAR-T (unpaired student *t*-test, two-tailed). F-CAR-T FasT CAR-T, C-CAR-T conventional CAR-T, Tcm (CD45RO+CD62L+) T central memory cells, Tem (CD45RO+CD62L−) T effector memory cells, Tscm (CD45RO−CD62L+) T stem cell memory, PD1 programmed cell death protein 1, TIM-3 T cell immunoglobulin and mucin domain containing-3, LAG3 lymphocyte-activation gene 3, RTCA real-time cell analyzer, E:T effector cells: target cells, NT normal T-cell.

To characterize the in vitro proliferative capacity of F-CAR-T cells, F-CAR-T and C-CAR-T cells were manufactured in parallel (Supplementary Methods, and Fig. S1) using T-cells from 6 B-ALL patients. To investigate the ex vivo proliferation of F-CAR-T, frozen CD19 F-CAR-T and C-CAR-T cells from each patient were thawed and stimulated with irradiated CD19-expressing K562 cells. The number of CD19-targeting CAR-T cells was then determined during the course of cell expansion in vitro. As shown in Fig. 1A, upon CD19 antigen stimulation, F-CAR-T proliferation was much more robust compared to C-CAR-T proliferation. On day 17 post co-culture, F-CAR-T expanded 1205.6 ± 1226.3 fold (Mean ± SD), while C-CAR-T expanded only 116.4 ± 37.2 fold (Mean ± SD), (*p* = 0.001). To characterize the mechanism underlying the superior proliferative ability of F-CAR-T, we purified CD19+ CAR-T cells from both F-CAR-T and C-CAR-T. The expression of genes involved in cell proliferation, cell cycle, and apoptosis was analyzed using Nanostring (detailed gene sets are in Table S2). Gene expression profiles showed higher F-CAR-T expression scores for genes associated with cell cycle regulation (F-CAR-T vs. C-CAR-T, *p* < 0.01) and lower expression scores for apoptosis-related genes (F-CAR-T vs. C-CAR-T, *p* < 0.05) in F-CAR-T cells (Fig. S2A).

#### F-CAR-T cells are less differentiated and less exhausted

Phenotypes of unstimulated F-CAR-T from three healthy donors were analyzed by flow cytometry. The CD45RO−CD62L+ population was 45.7% ± 2.2% which was comparable to the un-transduced T-cells (data not shown). Upon stimulation with CD19+ tumor cells for 9 days, C-CAR-T central memory cells (Tcm, CD45RO+CD62L+ and effector memory cells (Tem, CD45RO+CD62L−) were 56.62% ± 11.97% and 40.48% ± 9.70%, respectively, among the C-CAR-T cells (Fig. 1B and Figs. S2B and S2). In contrast, Tcm cells (87.92% ± 4.36%) was predominant in F-CAR-T, with only a small fraction of Tem (7.84% ± 3.79%). In addition, F-CAR-T cells demonstrated more abundant T stem cell memory (Tscm) (3.84 ± 1.22% vs 2.34 ± 2.48%, *p* < 0.05) than C-CAR-T cells. We also examined the exhaustion status of the stimulated CAR-T cells. A higher percentage of PD-1+LAG3+Tim3+ T-cells were detected in the C-CAR-T (11.19% ± 2.54%) compared to F-CAR-T (3.59% ± 2.51%, *p* < 0.001) (Fig. 1C). Together these data indicated that the F-CAR-T exhibited a “younger” phenotype and was less exhausted compared to C-CAR-T.

#### Potent in vivo killing capacity of F-CAR-T with improved persistence

We used a real-time cell analyzer (RTCA) assay to measure the cytotoxicity of F-CAR-T and C-CAR-T against CD19+ cells in vitro. F-CAR-T and C-CAR-T killing of Hela-CD19 target cells were comparable using this assay (Fig. 1D). Similar levels of IFN-γ and IL-2 production were also observed (Fig. S2D). In a luciferase-based cytotoxicity assay, CD19+ B leukemia cell lines, Raji and Nalm6, were both effectively killed to similar or better levels at different E:T ratios (Fig. 1E, F).

**Fig. 2:**
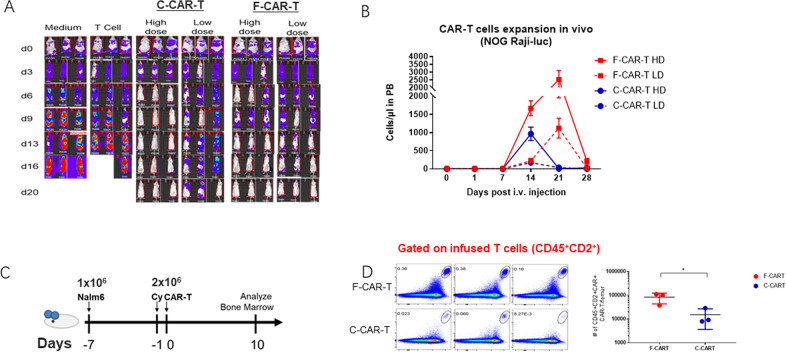
F-CAR-T show superior killing capacity and bone marrow migration in vivo. **A** Raji-Luc cell engraftment NOG mice were given high dose (2 × 10^6^/mice, *n* = 3) and low dose (5 × 10^5^/mice, *n* = 3) F-CAR-T/C-CAR-T along with control groups. Tumor growth was monitored with IVIS scan once every 3 days; **B** CAR-T expansion in peripheral blood of mice was analyzed by flow cytometry (*n* = 6). ***P < 0.001 for F-CAR-T HD vs. C-CAR-T HD; F-CAR-T LD vs. C-CAR-T LD; F-CAR-T HD vs. F-CAR-T LD; C-CAR-T HD vs. C-CAR-T LD (two-way ANOVA statistical analysis); **C** Schematic of the Nalm6 (1 × 10^6^) xenograft model, CAR-T (2 × 10^6^) infused 1 day after cyclophosphamide (20 mg/kg) treatment. Bone marrow infiltration of F-CAR-T was analyzed 10 days after CAR-T infusion (*n* = 3); **D** CD45^+^CD2 F-CAR-T vs. C-CAR-T in peripheral blood of mice were analyzed by flow cytometry; *P < 0.05 (unpaired student two-tailed *t*-test). IVIS in vivo imaging system, PB peripheral blood, i.v. intravenous, HD high dose, LD low dose, Cy cyclophosphamide; *p < 0.05; #: number.

To compare the in vivo cytotoxicity of F-CAR-T and C-CAR-T, severe immunodeficient NOG mice were engrafted with Raji-luciferase cells. One week after the tumor grafts were established, F-CAR-T and C-CAR-T were intravenously injected at various doses. The engrafted tumors progressed aggressively in control groups with either vehicle alone or control T-cells (Fig. 2A). In contrast, F-CAR-T or C-CAR-T treatment greatly suppressed tumor growth in a dose-dependent manner (Fig. 2A). In the high dose group (2 × 10^6^/mice), both F-CAR-T and C-CAR-T eliminated the tumor rapidly. However, in the low dose group (5 × 10^5^/mice), F-CAR-T showed more effective tumor-killing compared to C-CAR-T. On day 20, mice in the low dose F-CAR-T group became tumor-free, while C-CAR-T treated mice exhibited tumor relapse (Fig. 2A). We examined the CAR-T cell expansion in vivo after infusion. As shown in Fig. 2B, both F-CAR-T and C-CAR-T began to expand in the peripheral blood 7 days after infusion. C-CAR-T cell numbers reached their peak on day 14 and receded on day 21. In contrast, the F-CAR-T cell number peaked on day 21 and declined to a baseline level on day 28. F-CAR-T not only persisted longer but also underwent 2–6 folds greater expansion than C-CAR-T (Fig. 2B).

#### Increased BM migration of F-CAR-T

We examined the BM infiltration of F-CAR-T cells after infusion into Nalm6-bearing mice (Fig. 2C). A larger population of CAR-T cells was observed 10 days after infusion in BM in F-CAR-T infused group than that in the C-CAR-T group (*p* < 0.05) (Fig. 2D), suggesting F-CAR-T cells possessed a better BM homing capability than C-CAR-T.

The chemokine receptor CXCR4 is known to be critical for BM homing of T-cells [25, 26]. Indeed, a higher percentage of CXCR4+ T cells were detected in F-CAR-T than in the C-CAR-T. Interestingly, this phenotype was more pronounced for CD4+ T cells than CD8+ T cells (Fig. S3A). In a two-chamber system, more F-CAR-T cells could be detected in the lower chamber than their C-CAR-T counterparts (Fig. S3B).

### Clinical evaluation of FasT CAR-T cells

#### Clinical characteristics of enrolled patients

Between Jan. 2019 and Oct. 2019, 25 pediatric and adult patients with CD19+ R/R B-ALL were enrolled onto our phase 1 trial, including two patients who had relapsed following a prior allo-HSCT. Patient characteristics are detailed in Table 1. The median age of patients was 20 (range: 3–44) years old. Twenty patients were >14 years old, and five were ≤14 years old. The median percentage of pre-treatment BM blasts was 9.05% (range: 0.19–82.9%). As our pre-clinical studies demonstrated that F-CAR-T cells had a superior expansion capability as compared to C-CAR-T, we infused a relatively low doses of F-CAR-T cells, ranging from 10^4^–10^5^ cells/kg: 3.0 × 10^4^ cells/kg (*n* = 2), 6.5 (5.86–7.43) × 10^4^ cells/kg (*n* = 9), 1.01 (1.0–1.16) × 10^5^ cells/kg (*n* = 12), 1.52(1.47–1.56) × 10^5^ cells/kg (*n* = 2), (Fig. S4). The median time from apheresis to the infusion of CD19+ F-CAR-T cells was 14 days (range: 12–20). Although the manufacturing time of F-CAR-T was next day, the quality control time and detailed final product releases including sterility testing require a minimum of 7–10 days to complete. In addition, transportation of cell products requires approximately two days. Of the 25 patients who received CD19 F-CAR-T infusion, 22 (88%) received bridging chemotherapy between apheresis and lymphodepleting chemotherapy to control rapid disease progression (Table S3).

**Table 1:** Patient demographics and clinical outcomes.

Pt #	Gender (M/F)	Age (yrs)	BM blasts (%)	Disease status	Previous lines of treatment	Fusion gene/gene mutations	With prior transplant	Cell dose (CAR-T/kg)	Response 2 weeks	Response 4 weeks	CRS	ICANS
F01	F	15	0.19	Relapsed	4		No	1.07 × 10^5^	MRD − CRi	MRD − CRi	1	0
F02	M	4	0.2	Relapsed	1		No	7.43 × 10^4^	MRD − CRi	MRD − CR	3	4
F03	F	6	0.4	Relapsed	9		No	1.47 × 10^5^	MRD − CRi	MRD − CRi	3	4
F04	M	17	1.33	Relapsed	4	ERT	Yes	1.56 × 10^5^	MRD − CRi	MRD − CRi	2	0
F05	F	3	16.92	Relapsed	9	NRAS, CREBBP, TP53	No	3.0 × 10^4^	MRD − CRi	MRD − CR	3	4
F06	M	36	1.59	Refractory	5	BCR/ABL	No	6.62 × 10^4^	MRD − CR	MRD − CR	1	0
F07	F	5	0.31	Relapsed	14	CREBBP, NT5C2	No	3.0 × 10^4^	MRD − CRi	MRD − CRi	4	4
F08	M	21	9.59	Relapsed	3		No	7.04 × 10^4^	MRD − CRi	MRD − CR	3	0
F09	F	36	3.15	Refractory	4	MLL-AF4	No	1.00 × 10^5^	MRD − CRi	MRD − CRi	2	0
F10	F	20	0.38	Refractory	2		No	5.86×10^4^	MRD − CRi	MRD − CR	1	0
F11	M	39	0.41	Relapsed	8	BCR/ABL	No	1.16×10^5^	MRD − CRi	MRD − CR	3	0
F12	M	31	19.36	Relapsed	2	IKZF1	No	6.50 × 10^4^	MRD − CR	MRD − CR	1	3
F13	F	12	0.3	Relapsed	5		No	1.02 × 10^5^	MRD − CRi	MRD − CRi	2	4
F14	M	19	22.11	Relapsed	10		No	6.35 × 10^4^	MRD − CRi	MRD − CR	1	0
F15	M	19	9.05	Relapsed	8		No	6.00 × 10^4^	MRD + CRi	MRD + CR	1	0
F16	F	23	82.88	Relapsed	5	IKZF1	Yes	0.99 × 10^5^	MRD − CRi	MRD − CRi	1	0
F17	M	28	32.53	Relapsed	3		No	6.8 × 10^4^	MRD − CRi	MRD − CRi	1	3
F18	M	14	11.99	Relapsed	4	TP53	No	1.03 × 10^5^	MRD − CRi	MRD − CR	1	0
F19	F	34	9.40	Refractory	2		No	1.02 × 10^5^	MRD − CRi	MRD − CRi	2	0
F20	M	44	1.05	Refractory	2		No	1.03 × 10^5^	MRD − CRi	MRD − CRi	1	0
F21	F	18	22.48	Relapsed	4		No	1.0 × 10^5^	MRD − CRi	MRD − CRi	1	0
F22	M	17	14.59	Refractory	3	IKZF1	No	1.0 × 10^5^	MRD + CRi	MRD + CRi	0	0
F23	F	37	56.33	Relapsed	4	MLL-AF4	No	1.0 × 10^5^	MRD − CRi	MRD − CRi	1	0
F24	F	27	0.15	Refractory	3	BCR/ABL	No	6.0 × 10^4^	MRD − CRi	MRD − CR	1	0
F25	M	23	35.38	Relapsed	22		No	1.0 × 10^5^	MRD − CRi	MRD − CRi	3	0

*pt* patient, *M* male, *F* female, *BM* bone marrow, *CRS* cytokine release syndrome, *ICANS* immune effector cell-associated neurotoxicity syndrome, *MRD* minimal residual disease, *CR* complete remission, *CRi* CR with incomplete blood count recovery.

#### Characteristics of clinical manufactured F-CAR-T cells

F-CAR-T cells were manufactured successfully for all patients. The mean transduction efficiency of F-CAR-T was 35.4% (range: 13.1–70.3%) (Fig. S5A). Both CD4+/CAR+ (mean, 49.6%; range: 13.6–73.2%) and CD8+/CAR+ (mean, 41.5%; range: 20.6–77.7%) subsets were present in the CD3+ CAR+ T cell subsets of all products. The mean proportion of Tscm, Tem, and Tcm cells in the CD3+ CAR+ T cell subsets of all products was 23.3% (range: 3.55–45.3%), 33.2% (range: 17.2–67.9%), and 36.1% (range: 20.7–58.1%), respectively (Fig. S5B). F-CAR-T products exerted significant IFN-γ release and cytotoxic effects against the CD19+ cell line HELA-CD19 (Fig. S5, C, D).

#### Safety

All 25 infused patients experienced adverse events (AEs) of any grade, with 25 (100%) experiencing grade 3 or higher adverse events. No grade 5 events related to F-CAR-T treatment were observed (Table 2).

**Table 2:** Adverse events, CRS, and ICANS.

Variable	Total (*N* = 25)		
	Any grade	Grade 1–2	Grade ≥3
	number of patients (percent)		
Adverse events^a^			
Any	25 (100)	25 (100)	25 (100)
Hematological			
Leukopenia	22 (88)	0	22 (88)
Anemia	19 (76)	6 (24)	13 (52)
Lymphopenia	19 (76)	0	19 (76)
Thrombocytopenia	19 (76)	5 (20)	14 (56)
Neutropenia	16 (64)	2 (8)	14 (56)
Gastrointestinal			
Diarrhea	9 (36)	9 (36)	0
Nausea	8 (32)	8 (32)	0
Other			
Anorexia	13 (52)	13 (52)	0
Hypokalemia	12 (48)	10 (40)	2 (8)
Hypocalcemia	12 (48)	12 (48)	0
Fatigue	11 (44)	11 (44)	0
Hypoalbuminemia	9 (36)	8 (32)	1 (4)
Hyponatremia	9 (36)	9 (36)	0
Headache	9 (36)	9 (36)	0
Fever	8 (32)	8 (32)	0
Hypomagnesemia	7 (28)	7 (28)	0
Elevated ALT	6 (24)	6 (24)	0
Total bile acid increased	5 (20)	4 (16)	1 (4)
Hypophosphatemia	5 (20)	5 (20)	0
CRS	24 (96)	18 (72)	6 (24)
ICANS	7 (28)	0	7 (28)

^a^Listed adverse events are not designated as symptoms of CRS or ICANS and occurred in at least 20% of patients.

*ALT* alanine transaminase, *AST* aspartate transaminase, *CRS* cytokine release syndrome, *ICANS* immune effector cell-associated neurotoxicity syndrome.

CRS occurred in 24 (96%) patients with 18 (72%) grade 1–2 CRS,6 (24%) of grade 3, and no grade 4 or higher CRS (Fig. S6). In the >14 years old group, 16/20 (80%) patients developed mild CRS, and only 2/20 (10%) developed grade 3 CRS. For ≤14 years old patients, 2/5 (40%) had mild CRS, yet 3/5 (60%) experienced grade 3 CRS (Table S4). ICANS was observed in 7 (28%) patients, with 2 (8%) grade 3 ICANS occurring in patients >14 years old and 5 (20%) grade 4 ICANS all occurring in patients ≤14 years old. No grade 5 ICANS was developed (Fig. S7 and Table S4). The most frequent presentation of CRS was fever, particularly a high fever of >39 °C. The first onset of CRS symptoms occurred between day 3 and 8 post-CAR-T infusion with a median onset at day 4 (range: 1–10 days). The most common symptoms of ICANS were seizure (5/7) and depressed consciousness (5/7). The median time to ICANS onset from CAR-T cell infusion was 7 days (range: 5–8), and the median time to resolution was 2 days (Fig. S7). All CRS and ICANS events were managed including early intervention when fever of ≥39 °C persisted for 24 h. Sixteen (64%) patients received tocilizumab with a median total dose of 160 mg (range: 160–320 mg). Twenty-one (84%) patients received corticosteroids including dexamethasone (median total dose, 43 mg; range: 4–127 mg) and or methylprednisolone (median total dose, 190 mg; range: 40–1070 mg). The vast majority of these patients discontinued corticosteroids within 2 weeks. The change in IL-6, IFN-γ, IL-10, and GM-CSF levels after infusion are selectively shown in Fig. S8. The peak levels of these four cytokines were observed between day 7–10. Among all 21 cytokines examined, only post-infusion IL-6 levels were associated with moderate to severe CRS and/or ICANS (Figs. S9 and S10).

#### Cellular kinetics of F-CAR-T after infusion

**Fig. 3:**
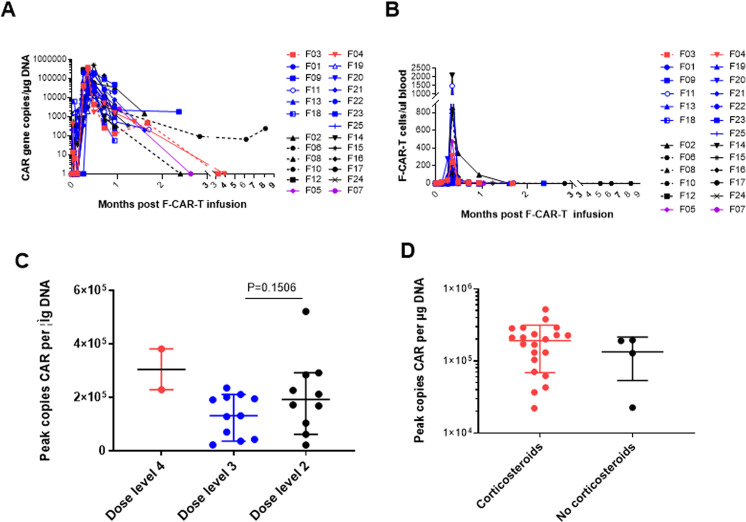
Cellular kinetics of F-CAR-T in patients. **A** F-CAR-T cells in peripheral blood by qPCR. Purple, dose level 1; black, dose level 2; blue, dose level 3; red, dose level 4; **B** F-CAR-T cells in peripheral blood by flow cytometry. Purple, dose level 1; black, dose level 2; blue, dose level 3; red, dose level 4; **C** Comparison of the mean peak copy number of F-CAR-T cells in peripheral blood at each dose level. Statistical significance was determined by the Mann–Whitney test. **D** Comparison of the mean peak copy number of F-CAR-T cells in peripheral blood with or without steroids. Statistical significance was determined by the Mann–Whitney test.

Superior in vivo proliferation and persistence of F-CAR-T compared to C-CAR-T cells were observed regardless of dose levels. The median peak level was reached on day 10 (range: 7–14 days) with 1.9 × 10^5^ transgene copies/µg of genomic DNA (range: 0.22–5.2 × 10^5^ transgene copies/µg of genomic DNA) by qPCR and 83 F-CAR-T cells per μl blood (range: 4–2102 F-CAR-T cells per μl blood) by FCM (Fig. 3A, B). No significant differences were observed among the different dose groups in the mean F-CAR-T copies peak (Fig. 3C). Importantly, there was no significant difference in the mean F-CAR-T copies peak between patients who received corticosteroids compared to those who did not (Fig. 3D).

#### Clinical response and duration of remission

**Fig. 4:**
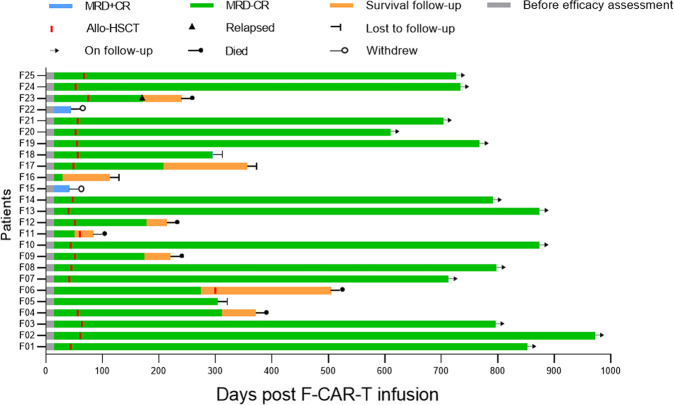
Clinical outcomes of patients after F-CAR-T infusion. Clinical outcomes and consolidative allo-HSCT for the 25 patients who were treated with F-CAR-T therapy are shown. On day 28, 23/25 patients achieved MRD-negative CR/CRi. With a median time of 54 days (range: 45–81) post F-CAR-T infusion, 20 of 23 patients with MRD-negative status received consolidative allo-HSCT. Among the 20 patients, 1 patient (F23) relapsed on day 172 and died 3 months after relapse. Four patients (F04, F09, F11, F12) died from transplant-related mortality (TRM) including infection (*n* = 3) and chronic GVHD (*n* = 1) on day 84, day 215, day 220, and day 312, respectively. The remaining 15 patients were in MRD-negative CR except for one (F18) who became MRD-positive on day 294. Among the other 3 patients (F05, F06, F16), 1 remained MRD-negative CR on day 304, 1 remained in MRD-negative CR until day 303, received allo-HSCT, and subsequently died from an infection on day 505. One patient was lost to follow-up after day 114. MRD minimal residual disease, CR complete remission, Allo-HSCT allogeneic hematopoietic stem cell transplantation.

Fourteen days after F-CAR-T cell infusion, all patients achieved morphologic CR including 2/25 with CR and 23/25 CR with incomplete hematologic recovery (CRi), which further improved to 11/25 CR and 14/25 CRi 28 days post F-CAR-T (Table 1 and Fig. 4). More importantly, 23/25 (92%) had the minimal residual disease (MRD)-negative remission on day 14 and day 28 after F-CAR-T treatment. Patients achieving remission through CAR-T were given the option to proceed to allo-HSCT. With a median time of 54 days (range: 45–81 days) post F-CAR-T infusion, 20 of 23 patients with MRD-negative status decided to pursue consolidative allo-HSCT including one patient who received a 2nd transplant. As of 18 October 2021, with a median follow-up duration of 693 days (range: 84–973 days) among the 20 patients who had received allo-HSCT, one patient relapsed on day 172 and died 3 months after relapse, and four patients died from transplant-related mortality (TRM) including infection (*n* = 3) and chronic GVHD (*n* = 1) on day 84, day 215, day 220, and day 312, respectively. The other 15 patients remained in MRD-negative CR with a median remission duration of 734 days (range: 208–973) except for one who became MRD-positive on day 294 with CD19+ disease. Among the other three patients (F05, F06, F16), one remained in MRD-negative CR on day 304, one remained in MRD-negative CR until day 303, received allo-HSCT but died from an infection on day 505, and one was lost to follow-up after day 114. Two patients who had MRD-positive CR after infusion withdrew from the study on day 42 and day 44, respectively, to seek other studies.

#### Clinical association of F-CAR-T cell level, cytokines in cerebrospinal fluid (CSF), and PB

F-CAR-T/T ratio in cerebrospinal fluid (CSF) was evaluated by FCM in 13/25 patients with available samples (Table S5). Between days 10 and 32, 9 patients were found to have considerable F-CAR-T penetration in their CSF, ranging from 40.65 to 79.2%, including 4 who developed severe ICANS. Among the other 4 patients, F-CAR-T cell abundance in the CSF ranged from 1.29% to 3.57%, and none experienced severe ICANS. Patients with higher levels of CAR-T in PB on day 10 consistently had higher levels of CAR-T in CSF with the exception of patient F15. Notably, CAR-T cells were still detectable in the CSF on day 101 with a 2.36% CAR-T/T ratio in patient F06, who also had undetectable circulating CAR-T cells at the same time.

In addition, concentrations of seven cytokines (IL-1b, IL-6, IL-10, IFN-γ, TNF-α, MCP-1, and GM-CSF) in CSF samples from the above 10 of 13 patients were measured. Specifically, IL-1b was not detected in any of the 10 patients, and only one patient had detectable GM-CSF. For the other five cytokines, patients with severe ICANS had higher IL-6 levels in contrast to patients without severe ICANS, and the difference between the median level of IL-6 among these two groups of patients was statistically significant (Fig. S11). We did not observe significant differences among the other 4 cytokines between the two groups of patients. No clear relation between the CSF cytokine levels and the F-CAR-T/T % was observed.

## Discussion

While CD19-targeting CAR-T cell therapy has shown significant efficacy for B-ALL patients, the high cost and lengthy process of CAR-T production limit its broader use. In addition, its therapeutic potential in terms of depth and the duration of remission can still be further improved. Here, a new platform of FasT CAR-T with a shortened next-day manufacturing time was developed. Pre-clinical studies have demonstrated F-CAR-T to be less exhausted than conventional CAR-T with a superior proliferation. Our first-in-human clinical study demonstrated that CD19 F-CAR-T showed early promising efficacy with a manageable safety profile in both pediatric and adult B-ALL patients.

At present, anti-CD19 CAR-T takes an average of two weeks to manufacture and an additional 7–14 days to complete quality control testing [9–12]. The F-CAR-T procedure reported herein successfully shortens the manufacturing procedure from 7 to 14 days to next day, and in the current trial, had a 100% manufacturing success rate among heavily pretreated R/R B-ALL patients and those with highly aggressive disease. All 25 patients enrolled in our study received the therapy in a timely fashion with next-day manufacturing of the product and a median of 14 days from apheresis driven by 7 days of rapid sterility testing, which is a significant improvement from the median 45 days it takes from receiving the leukapheresis made in the manufacturing facility to infusion, as reported in ELIANA clinical trial [11]. In addition, our F-CAR-T manufacturing platform reduced the average hospital stays by about one week prior to F-CAR-T infusion and has the potential to decrease the length of and the need for bridging chemotherapy that is often necessary to control rapid disease progression.

This novel procedure additionally affords several significant improvements over C-CAR-T including superior expansion capability, higher levels of Tcm and Tscm cells, less exhausted T-cells, better penetration to the BM and greater effectiveness in eliminating B-ALL in a xenograft mouse model. These characteristics of F-CAR-T presented in our preclinical study likely contribute to the rapid clinical response observed in our clinical study. Our preclinical study indicates that Tscm and Tcm are enriched in the FasT CAR-T population, partially explaining why F-CAR-T expands 10-fold greater in vitro and twofold more in vivo upon CD19 antigen stimulation compared to C-CAR-T. Gene expression profiling indicates that F-CAR-T expresses proliferation-related genes at higher levels and apoptosis-related genes at lower levels compared to C-CAR-T. F-CAR-T cells also express reduced levels of PD-1+ and LAG3+ and are less prone to exhaustion compared to C-CAR-T. Finally, CXCR4+ T cells are represented at a higher percentage in both CD4+ T and CD8+ T cell subsets of F-CAR-T, culminating in significantly higher homing and infiltration of F-CAR-T in BM. Altogether, these characteristics of F-CAR-T contribute to their superior in vivo killing capacity compared to C-CAR-T cells. In clinical trials of C-CAR-T for B-ALL, 4 weeks are often required to achieve a clinical response [9–11]. In contrast, 14 days after F-CAR-T infusion, 25/25 (100%) achieved CR/CRi and 23/25 (92%) had MRD-negative CR. The two patients who had MRD-positive CR on day 14 failed to convert into MRD-negative CR on day 28, suggesting maximal clinical remission benefit had already been achieved in 14 days.

Despite the initial high CR with C-CAR-T therapy, relapse remains a major problem and occurs in about 28–43% of patients with B-ALL after CAR-T treatment alone [9–11]. There is much debate on whether allo-HSCT consolidation is necessary after CAR-T therapy for B-ALL [5, 6, 9, 10]. Accumulating evidence indicates that consolidative allo-HSCT after CAR-T improves LFS/OS and should be considered, especially in high-risk ALL patients [20–22, 27]. As such, the option of pursuing consolidative allo-HSCT was given when patients achieved MRD-negative CR. With a median follow-up time of 693 days (range: 84–973 days), only one relapse was observed in patients (*n* = 20) who underwent allo-HSCT after F-CAR-T. However, 4/20 (20%) patients who pursued consolidative allo-HSCT died from TRM, comparable to the reported rate of between 23 and 38% [9, 21]. The majority of patients who responded to CD19 F-CAR-T therapy subsequently received consolidative allo-HSCT therapy which limited our ability to assess the durability of the clinical response to CD19+ F-CAR-T alone. However, this proof of concept first-in-human clinical study was designed to primarily evaluate the safety and feasibility of our Fast CAR-T manufacturing platform. It is particularly encouraging that two patients treated with F-CAR-T alone had at least ten months of leukemia-free at the last follow-up. Additional data with longer follow-up are needed to better understand the long-term safety and durability of CD19 F-CAR-T therapy alone.

The superior expansion capacity of F-CAR-T was confirmed in our clinical study with a peak between day 7 and day 10 by FCM or qPCR, and independent of the infused CAR-T dose. With robust F-CAR-T cell proliferation in vivo, an infusion of as low as 3.0–6.0 × 10^4^ cells/kg in our lower dose group (*n* = 11) resulted in CR in all patients treated. The ability of next-day manufactured F-CAR-T to control leukemia at a substantially lower dose supports our preclinical findings of the superiority of F-CAR-T over C-CAR-T cells. Our data also show that corticosteroids used for treating CRS and ICANS did not affect the CR rate nor did it appear to adversely affect the expansion/persistence of functional F-CAR-T cells, supporting the practice of early intervention for treating CRS as suggested by some studies [28–31]. In addition, despite the small dose of F-CAR-T cell infused, significant central nervous system (CNS) penetration was observed with as high as 79% of FasT CART cells among the T-cell population in the CSF of patients. Higher F-CAR-T levels in the PB on day 10 predicted this high level in the CSF. Good penetration of F-CAR-T in the CSF may be an important clinical advantage to reduce CNS relapse. On the other hand, it may also potentially increase CNS toxicities.

No death due to F-CAR-T treatment toxicity occurred in this study. The incidence of severe CRS was 24% and the incidence of ICANS was 28%. All five patients younger than 14 years old experienced severe ICANS as compared to patients old than 14 years old with only 2/20 severe ICANS. We observed that the median concentration of IL-6 in the CSF from patients with severe ICANS was significantly higher than that of patients without severe ICANS (23.9 versus 4.6), suggesting that IL-6 in CSF may play an important role in the development of severe ICANS, as previously reported [32–34]. We speculated that severe neurotoxicity might be associated with the increased blood-brain barrier permeability in pediatric patients that permits the transit of plasma cytokines and CAR-T cells into the CSF [32] together with the feature of our F-CAR-T of a higher and more rapid proliferation capacity of young CAR-T cells and CD28 co-stimulator. This may partly explain the higher incidence of ICANS observed among the pediatric patients in contrast with the incidence of severe ICANS among pediatric patients treated with other CAR-T products for B-ALL [7, 10]. We observed that the neurotoxicity was much lower among the pediatric patients treated with F-CAR-T with 4-1BB as a co-stimulator in a trial we subsequently conducted and reported at ASH 2020 [35]. Although an increased risk of severe ICANS was seen in patients younger than 14 years old, patients ≥ 14 years old experienced a good safety profile.

In summary, the present study has demonstrated that a robust, rapid cell production process of F-CAR-T cells is feasible and a sufficient number of cells can be generated the next day with superior expansion capability, more abundant Tscm/Tsm subsets, and less exhausted phenotypes compared to C-CAR-T cells. Our first-in-human clinical study has further indicated that our approach is safe, reliable, and highly effective for treating adolescent and adult patients with B-ALL, paving the road for expanding our F-CAR-T platform to other CAR-T products and conditions other than B-ALL and to antigens beyond CD19. Our FasT CAR-T manufacturing platform could represent a more cost-effective method to provide CAR-T cell immunotherapy to patients by significantly reducing both manufacturing time and cost, as well as decreasing patients’ clinical hospital stays.

## Supplementary information

supplementary materials

## Data Availability

To get a detailed protocol on the clinical trial, please find it at https://clinicaltrials.gov, NCT03825718. For any information regarding this manuscript, please contact the corresponding author at peihua_lu@126.com.
