# Supplementary material for: Next-day manufacture of a novel anti-CD19 CAR-T therapy for B-cell acute lymphoblastic leukemia: first-in-human clinical study
Source: Blood Cancer J. 2022 Jul 7;12(7):104. doi: 10.1038/s41408-022-00694-6 (PMC9262977; doi:10.1038/s41408-022-00694-6)
Supplement: Supplementary file 1 — supplementary materials [file 41408_2022_694_MOESM1_ESM.docx]

Supplementary materials for

**Next-Day Manufacture of a Novel Anti-CD19 CAR-T Therapy for B-Cell Acute Lymphoblastic Leukemia: First-in-Human Clinical Study**

Junfang Yang^1,2*^, Jiaping He^3*^, Xian Zhang^1,2*^, Jingjing Li^1,2^, Zhenguang Wang^3^, Yongliang Zhang^3^, Liyuan Qiu^1^, Qionglu Wu^3^, Zhe Sun^3^, Xun Ye^3^, Wenjie Yin^3^, Wei Cao^3^, Lianjun Shen^3#^, Martina Sersch^3^, Peihua Lu^1,2#^

**Affiliations:**

1. Hebei Yanda Lu Daopei Hospital, Langfang, Hebei

Address: Si Pu Lan Road, Yanjiao Economic and Technological Development Zone, Langfang, Hebei, 065201, China.

1. Beijing Lu Daopei Institute of Hematology, Beijing, China

Address: No.22 Tongji South Rd, Yizhuang Economic and Technological Development Zone, Daxing District, Beijing, 100176, China.

1. Gracell Biotechnologies Co., Ltd, Shanghai, China

Address: 926 Yishan Road, Xuhui District, Shanghai, 200233, China

***These authors contributed equally to this work.**

**# Corresponding author:**

Peihua Lu

Beijing Lu Daopei Institute of Hematology

No.22 Tongji South Rd, Yizhuang Economic and Technological Development Zone, Daxing District, Beijing, 100176, China

Email: peihua_lu@126.com

Phone: +861086841225; Fax: +861086841225

or Lianjun Shen

Gracell Biotechnologies Co., Ltd, Shanghai, China

Address: 926 Yishan Road, Xuhui District, Shanghai, 200233, China

Email: lj.shen@gracellbio.com

Phone: +862164031375

**This file includes:**

Supplementary Methods

Fig. S1. Schematic of CD19 FasT CAR construct and next-day manufacturing process.

Fig. S2. Characterization of F-CAR-T *in vitro*.

Fig. S3. F-CAR-T showed enhanced migration and increased CXCR4 expression.

Fig. S4. Patient screening, enrollment and infusion.

Fig. S5. Characteristics of clinical manufactured F-CAR-T cells.

Fig. S6. Presentation and outcomes of patients with CRS.

Fig. S7. Presentation and outcomes of patients with ICANS.

Fig. S8. Plasma cytokine levels after F-CAR-T infusion.

Fig. S9. Association of cytokines post infusion with severe CRS and ICANS

Fig. S10. Association of cytokines before infusion with severe CRS and ICANS

Fig. S11 Comparison of the cytokines in CSF between patients with or without severe ICANS.

Table S1. FasT CAR-T release test and criteria.

Table S2. Gene sets.

Table S3. Bridging chemotherapy prior to FasT CAR-T cells.

Table S4. CRS and ICANS are grouped by age.

Table S5. Clinical correlations with cytokines of CSF and PB.

Table S6. Flow antibody list.

## Supplementary Methods

**Preclinical section**

***CD19 CAR construct***

The mouse FMC63 anti-CD19 scFv was inserted into a second-generation CAR cassette containing a CD8a signaling peptide, a CD8a hinge region, CD8a transmembrane domain, CD28 costimulatory domain, and CD3ζ activation domain (Figure S1A). A FLAG tag (DYKDDDDK) (termed Beacon) was inserted into the CD19 CAR between the scFv and the hinge region for the purpose of detection by flow cytometry. This CAR construct is herein called the CD19 CAR.

***Cell lines***

K562 (human chronic myelogenous leukemia) (ATCC, CCL-243™), Raji (B cell Burkitt's lymphoma) (ATCC, CCL-86™), NALM6 (B cell leukemia) (ATCC CRL-3273), HeLa (ATCC, CCL-2) were purchased from ATCC. HeLa cells were cultured in DMEM and other cell lines were cultured and maintained in RPMI-1640 (Invitrogen, Carlsbad, CA, USA), both supplemented with 10% heat inactivated FBS (Invitrogen) at 37°C, 5% CO2. CD19-expressing K562 (K562-CD19) and HeLa (HeLa-CD19) were generated by lentiviral transduction with vector purchased from Genechem (Shanghai, China). To evaluate tumor killing in vitro and in vivo, Raji and NALM6 cells were also transduced with lentivirus for luciferase purchased from Genechem (Shanghai, China).

***Cell proliferation assay***

To evaluate *ex vivo* proliferation of F-CAR-T cell, frozen F-CAR-T and C-CAR-T cells were thawed and grown in cell culture. Irradiated K562-CD19 cell were added once every 3 days to stimulate CAR-T cells expansion. During the expansion, cultured CAR-T cell were aliquoted and cell viability and number were determined using the NC-200™ Automated Cell Counter. The total number of CAR+T cells undergoing expansion was calculated based on the CAR+ T% determined by flow cytometry. The fold of expansion was determined as [CAR-T number at indicated day] / [CAR-T number at day 0].

***Cytotoxicity assay***

Cytotoxicity assays were performed on an xCELLigence RTCA instrument (ACEA Biosciences, USA) according to the manufacturer’s instructions. Briefly, HeLa-CD19 cells were seeded at 5,000 cells per well in a 96-well plate. On the next day, human CAR T cells were re-suspended in fresh complete medium without IL-2 were incubated with target cells at indicated E/T ratios and killing of Hela-CD19 cells were determined as per manufacturer’s instructions. Cytotoxicity assay was also performed after CAR-T effector cells and luciferase expressing Raji or Nalm6 target cells were co-cultured for 6 hours. Target cell killing was evaluated by luciferase activity, assayed with the Promega One-Glo Luciferase Assay System.

***Flow cytometry***

Blood samples were collected from patients or experimental animals. Erythrocytes were lysed with red blood cell lysing medium (BD Bioscience, #349202) and white blood cells were centrifuged and washed with 2ml of phosphate buffered solution (PBS) before staining. Human CAR-T cells were identified with surface staining of CD45, CD2, and CAR expression were detected using an anti-FLAG antibody. For immunophenotyping, F-CAR-T and C-CAR-T cells were harvested, washed twice with 2 ml PBS, and stained with flow cytometry-grade antibodies (Table S6). All flow antibodies were titrated before use, and fluorescence-minus-one (FMO) controls were created for each antibody panel to set gates for positive events. 7AAD and absolute counting beads were added before flow cytometry was performed using FlowCanto. Flow cytometry data were analyzed using the FlowJo software. Cytometric beads array (CBA) was used to measure mouse serum cytokines. Flow cytometry was used to detect CAR expression, differentiation status of the manufactured CAR-T cells, and CAR-T cell levels in the cerebrospinal fluid (CSF) and peripheral blood (PB).

***Quantitative PCR***

Genomic DNA (gDNA) was extracted from cells in 1 ml of whole blood using the QIAamp DNA Blood Midi kit (Qiagen, Redwood City, CA, United States, 51185). The gDNA was used as the template to detect the integrated CAR-T lentivirus vector using the TB Green Premix ExTaq (Tli RnaseH Plus) Kit (Takara Biotechnology, Shiga, Japan, RR420A). Real-time PCR amplification was carried out using the Applied Biosystems 7500 Real-Time PCR System (Life Technologies, MA, USA). A primer pair targeting the WPRE region was used to determine CAR copy number and a primer pair targeting the cellular RPP30 gene was used as an internal control. The primer pairs were experimentally validated using the following criteria: (i) a single gene-specific product was produced; (ii) the amplification efficiency ranged between 90% and 110%; and (iii) the cycle threshold (Ct) value of the no-template DNA control was more than 40.

***Cytokine assay***

From RTCA cytotoxicity assay, cell supernatant was harvested and frozen after centrifugation. IFNγ and IL-2 concentrations were measured using microfluidic simple plex cartridge-based ELISA assay on Ella (Protein simple, CA).

To detect serum cytokine production in CAR-T infused patient, human blood sample were collected. After centrifugation to remove cells, serum sample was aliquoted and frozen at −80°C immediately. Cytokines were analyzed using the Luminex 200 apparatus (Luminex Corporation, MN, USA). The following cytokines, chemokines and growth factors were measured in the cohort samples: IL-2, IL-6, IL-7, IL-8, IL-10, IL-12p70, IL-15, tumor necrosis factor (TNF) α, granulocyte-macrophage colony-stimulating factor (GM-CSF), interferon (INF) γ, granzyme B, CCL19, MCP-1, vWF-A2, Angiopoietin-1, and Angiopoietin-2.

Cytokine levels in CSF were determined using the Cytometric Beads Array kit (CBA, QuantoBio company, China) to quantify human IFNγ, IL1β, IL2, IL4, IL5, IL6, IL8, IL10, IL-12p70, IL-17A, IL-17F, IL22, TNFα, TNFβ, sCD25, GM-CSF, IL-15, CCL2, Granzyme B, Reg3A, IL-1R4, sCD120a, Elafin and CCL3. The CBA immunoassay in thawed peripheral blood (PB) supernatants or CSF was carried out according to the manufacturer’s protocols.

***Transwell assay***

Cell migration assays were performed using 24-well microchemotaxis chambers (Costar, CLS3421-48EA) with uncoated polycarbonate membranes (pore size 5 μm). Briefly, mSDF-1α (R&D system, Cat.#.460-SD) was diluted to the indicated concentration and loaded in the bottom chamber. CAR-T cells were harvested and labeled with CFSE, and 5x10^4^ cells in 0.1 mL of ex vivo T cell culture medium were seeded in the top chamber. The plates were incubated for 4h at 37°C. The cells that migrated through the filter were photographed using the fluorescence microscopy at 20x magnification and fluorescent cells counted with ImageJ software.

***In vivo migration assay***

1x10^6^ Nalm6 cells in 200ul PBS were injected i.v into NOG mice. Seven days later, tumor bearing mice were infused with 2x10^6^ F-CAR-T or C-CAR-T for 1 day and then treated with 20 mg/kg cyclophosphamide (CTX). Ten days after CAR-T infusion, CAR-T appearance in bone marrow was determined by flow cytometry.

***Gene Expression Profiles***

F-CAR-T and C-CAR-T cells were co-cultured respectively with irradiated K562-CD19 at a 2:1 ratio. Six days after stimulation, CAR-T cells labeled with biotinylated-anti-Beacon were sorted with Streptavidin beads. RNA was extracted from purified CAR-T cells and evaluated for gene expression using the CAR T Characterization Gene Expression Panel (Nanostring, Seattle, WA), which detects 780 CAR-T biology related genes and associated controls. Gene analyses were carried out by WuxiAppTec (Shanghai, China). Reference gene normalization was performed for each sample by dividing each sample’s raw count profile by the geometric mean of its reference genes. To transform expression back to an intelligible count space, all samples were then multiplied by the geometric mean of all the samples’ reference gene geometric means. The nSolver software was used to perform all normalization. Cell scores were calculated as the average log2 normalized expression of each cell’s marker genes.

***Animal model***

Animal studies were carried out in Biocytogen (Beijing, China), CrownBio (Taicang, China) and JOINN Lab (Taicang, China). Female and/or male NOG mice at 8-12 weeks of age were used in accordance with protocols approved by the Institutional Animal Care and Use Committee (IACUC) of the Contract Research Organization (CRO) service providers.

For tumor killing efficacy study, NOG mice were injected i.v. with Raji-luc cell (3x10^5^/mouse), followed by i.v. infusion of various dose of CD19-targeted CAR-T cells (5x10^5^ or 2x10^6^ CAR-T cells/mouse, 3 mice/group) 7 days later. Tumor growth in Raji-luc-engrafted mice was monitored by bioluminescent imaging (BLI), Tumor burden were determined by quantitative analysis of bioluminescent signal intensity.

For *in vivo* CAR-T expansion study, NOG mice were injected i.v. with Raji-luc cell (3x10^5^/mouse), followed by i.v. infusion of various dose of CD19-targeted CAR-T cells (5x10^5^ or 2x10^6^ CAR T cells/mouse, 6 mice/group) 7 days later. CAR-T expansion in PB were analyzed by flow cytometry at indicated time.

**Clinical section**

***Assessment of clinical response***

 For the clinical study, anti-tumor response was initially assessed in all patients at 2- and 4-weeks post F-CAR-T cell infusion respectively. CR was defined as the presence of less than 5% bone marrow (BM) blasts, the absence of circulating blasts, and no extramedullary sites of disease as confirmed by position-emission tomography (PET)/computed tomography (CT), regardless of cell-count recovery. CR or CR with incomplete count recovery (CRi), relapse and minimal residual disease (MRD) were defined in accordance with the national comprehensive network (NCCN) guideline, version 1, 2018.  MRD-negative CR was defined as absence of leukemic blasts in the BM by high-resolution multiparameter flow cytometry (sensitivity 1:10,000).

**Supplementary Figures**

**Supplementary Figure Legends**

**
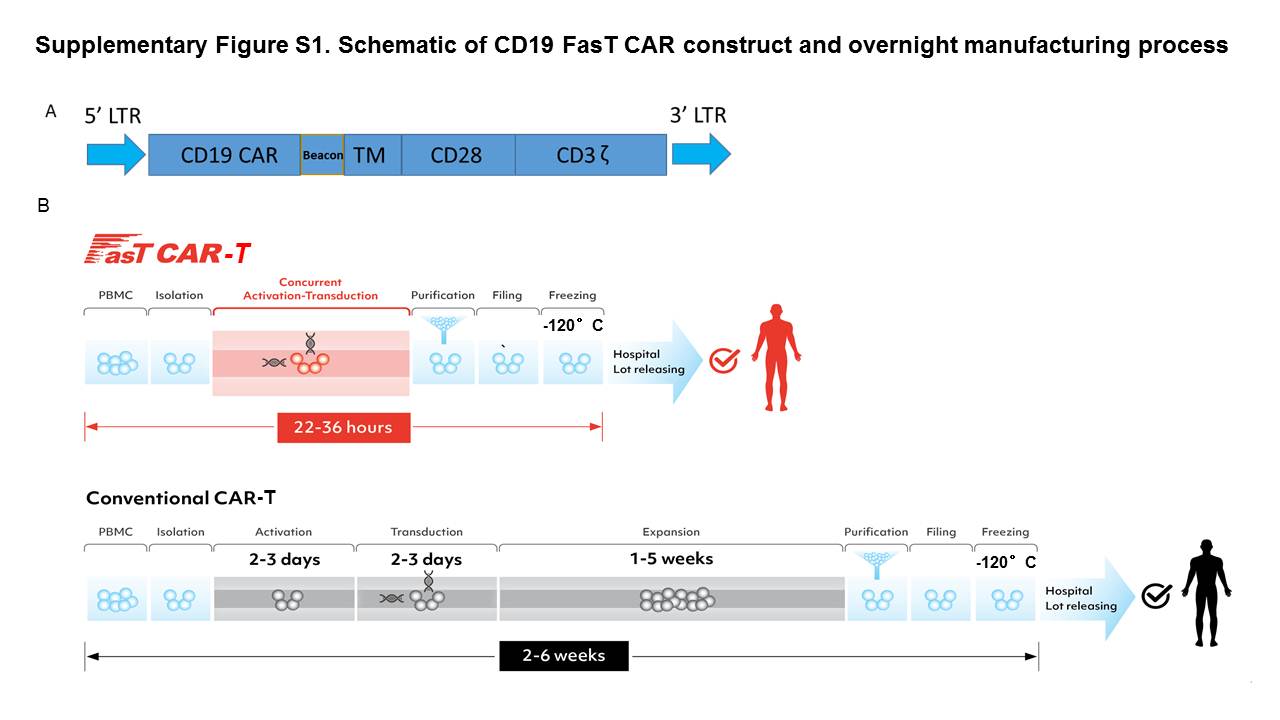
Supplementary Figure S1. Schematic of CD19 FasT CAR construct and next-day manufacturing process**

(A)Schematic illustration of CD19 FasT CAR construct. (B) Manufacture process of FasT CAR-T vs. conventional CAR-T. PBMCs were obtained from healthy donors and enrolled patients by leukapheresis. Isolated T cells were isolated using Dynabeads CD3/CD28 CTS and transduced next-day with CD19 CAR lentiviral vectors in X-vivo culture medium containing IL-2. F-CAR-T cells were collected the next day without expansion step while C-CAR-T cells required 1-5 weeks for expansion. F-CAR-T products were harvested and cryopreserved.

LTR: long terminal repeats; TM: transmembrane; PBMCs: peripheral blood mononuclear cells；CTS: Cell Therapy Systems; F-CAR-T: FasT CAR-T; C-CAR-T: Conventional CAR-T.

**
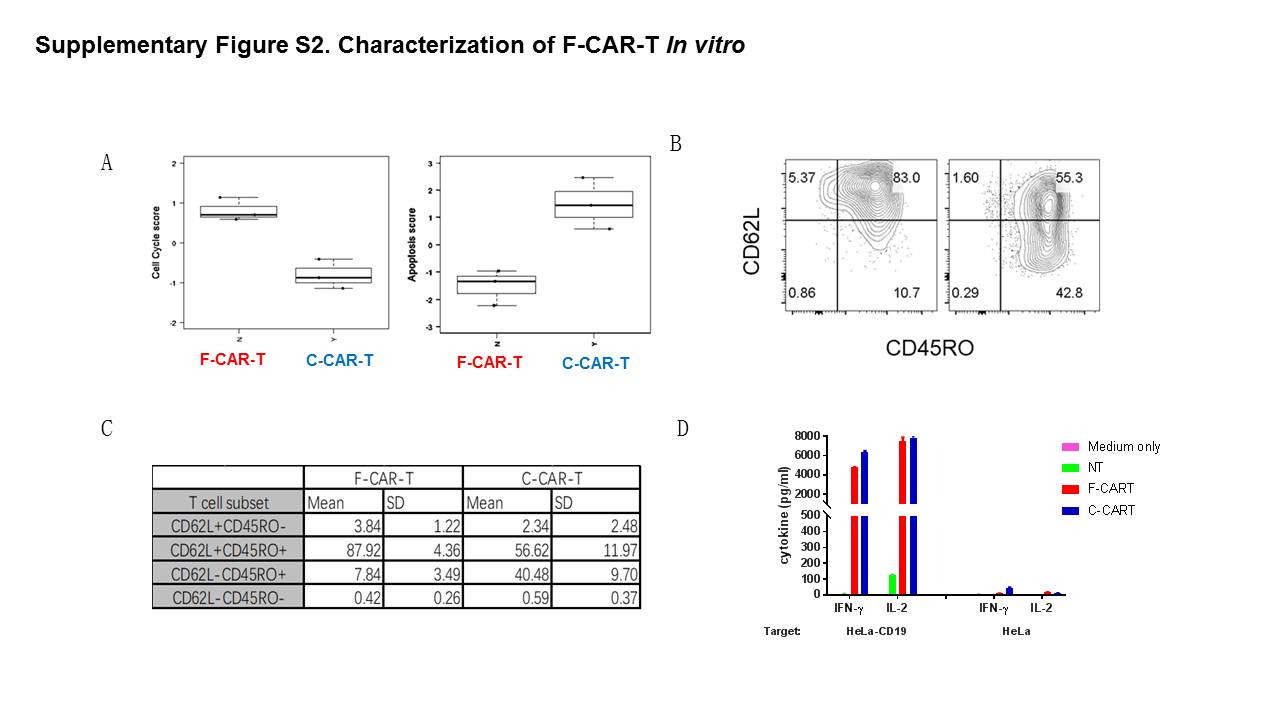
**

**Supplementary Figure S2. Characterization of F-CAR-T *In vitro***

(A)Transcriptional gene profile of purified F-CAR-T and C-CAR-T cells were compared in parallel. Represented data are cell cycle (F-CAR-T vs. C-CAR-T, **P<0.01) and apoptosis (F-CAR-T vs. C-CAR-T, *P<0.05) related gene set. Student *t* test was used for statistical analysis. (B) Tscm, Tcm and Tem were characterized by surface staining of CD45RO and CD62L and analyzed with flow cytometry. (C) Table summarizing memory phenotype of F-CAR-T and C-CAR-T cells. (D) Cytokine production from the supernatant was quantified using CBA flow assay.

CBA: Cytometric Bead Assay; Tcm (CD45RO+CD62L+): T central memory cells; Tem(CD45RO+CD62L-): T effector memory cells; Tscm(CD45RO-CD62L+): T stem cell memory; F-CAR-T: FasT CAR-T; C-CAR-T: Conventional CAR-T; NT: normal T cells.

**
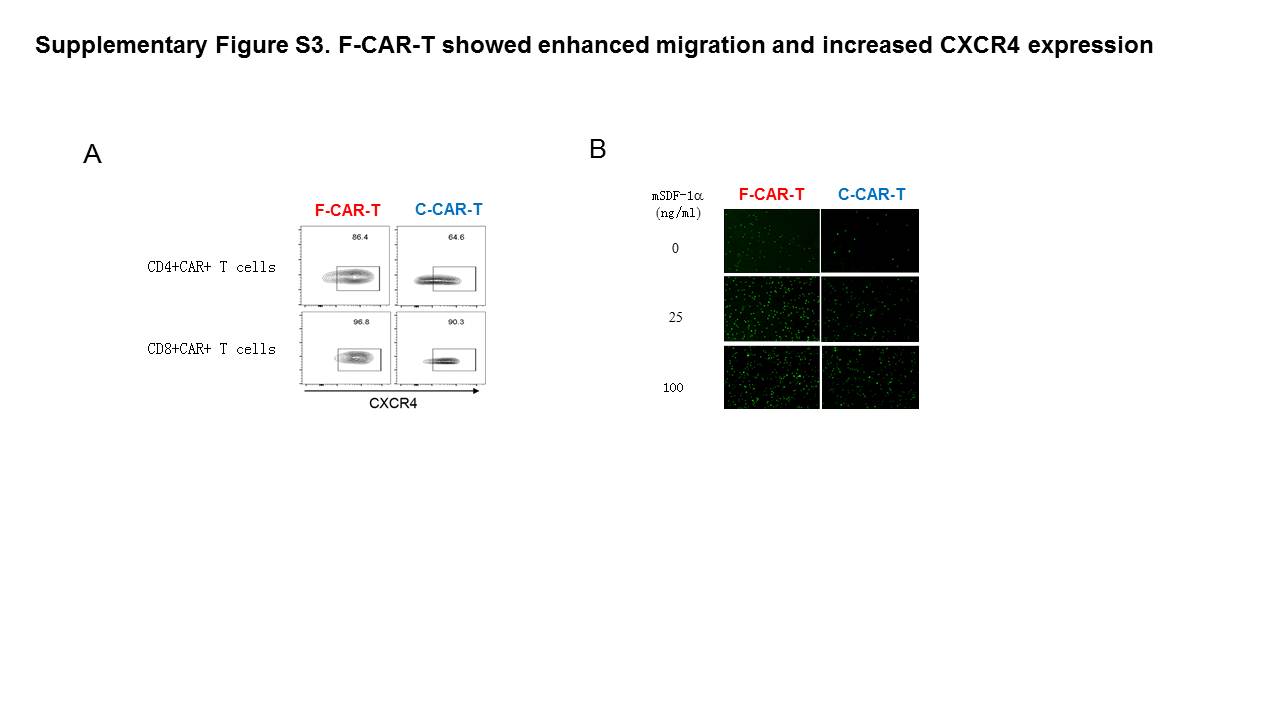
**

**Supplementary Figure S3. F-CAR-T showed enhanced migration and increased CXCR4 expression**

(A) Surface staining of CXCR4 on CD4+ and CD8+ CAR+ T cells. (B) Transwell migration assay demonstrating dose dependent migration of CFSE labeled F-CAR-T vs. C-CAR-T cells imaged using fluorescence microscopy.

CXCR4:C-X-C chemokine receptor type 4; CFSE: carboxyfluorescein diacetate, succinimidyl ester; F-CAR-T: FasT CAR-T; C-CAR-T: conventional CAR-T.

**
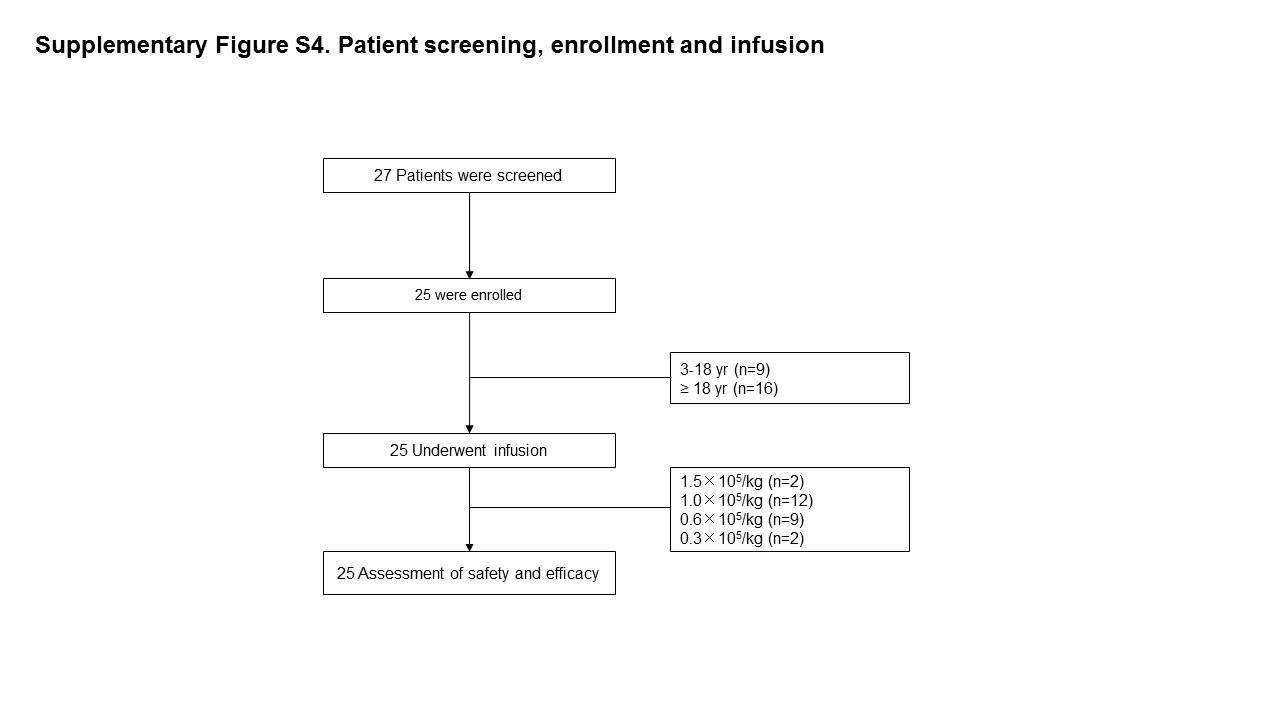
**

**Supplementary Figure S4**. **Patient screening, enrollment and infusion.**

Twenty-five patients were enrolled in the study. F-CAR-T cells were successfully manufactured and infused into the 25 patients at four different doses (1.5×10^5^/kg, n=2; 1.0×10^5^/kg, n=12; 0.6×10^5^/kg, n=9; 0.3×10^5^/kg, n=2). All the 25 patients were assessed for safety and efficacy post F-CAR-T cells infusion.

yr:years; F-CAR-T: FasT CAR-T; C-CAR-T: conventional CAR-T.


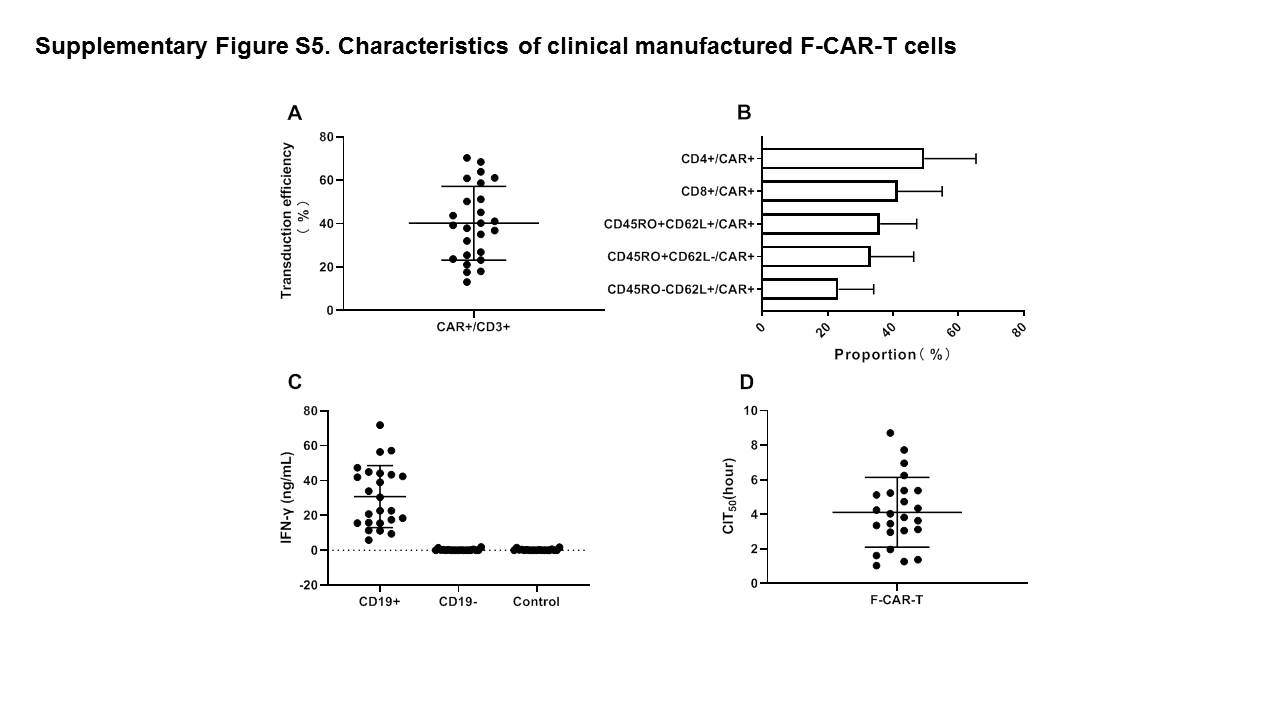


**Supplementary Figure S5. Characteristics of clinical manufactured F-CAR-T cells**

(A) CAR expression on F-CAR-T products. Individual data points and mean transduction efficiency of F-CAR-T are shown. (B) Phenotypic analysis of CAR positive T cells subset of F-CAR-T product. T Naïve: CD3+CD45RO-CD62L+; T effector memory cells: CD3+CD45RO+CD62L-; T central memory cells:CD3^+^/CD45RO^+^/CD62L^+^; Box plot with whiskers (tukey) is shown. (C) IFN-γ release assay using F-CAR-T as effectors and CD19 positive (Hela-CD19) and CD19 negative(molt-4/Hela) cell lines as targets. Effector to target ratio is 1:1. (D) RTCA analysis using F-CAR-T as effectors and CD19 positive (Hela-CD19) cell lines as targets. The Effector to target ratio is 2.5:1. Individual data points and mean is shown.

RTCA: real-time cell analyzer; IFN-γ: interferon gamma; CIT50: standard curve for the time for the cell index to decrease 50%.

**
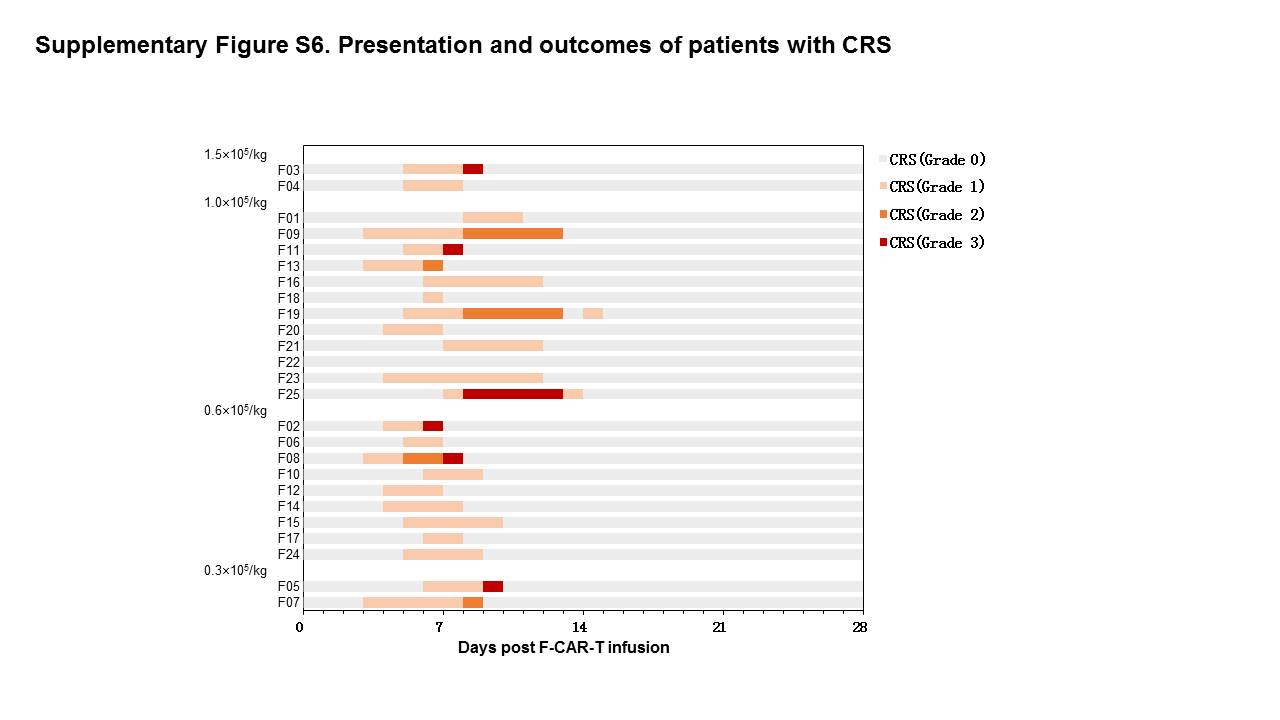
**

**Supplementary Figure S6. Presentation and outcomes of patients with CRS**

CRS was observed in 24 (96%) patients, with 18(72%) grade 1-2, 6 (24%) grade 3, and no grade 4 or higher. First onset of CRS symptoms occurred between day 3 to 8 post infusion with a median duration of 4 days (range, 1-10).

CRS: cytokine release syndrome.

**
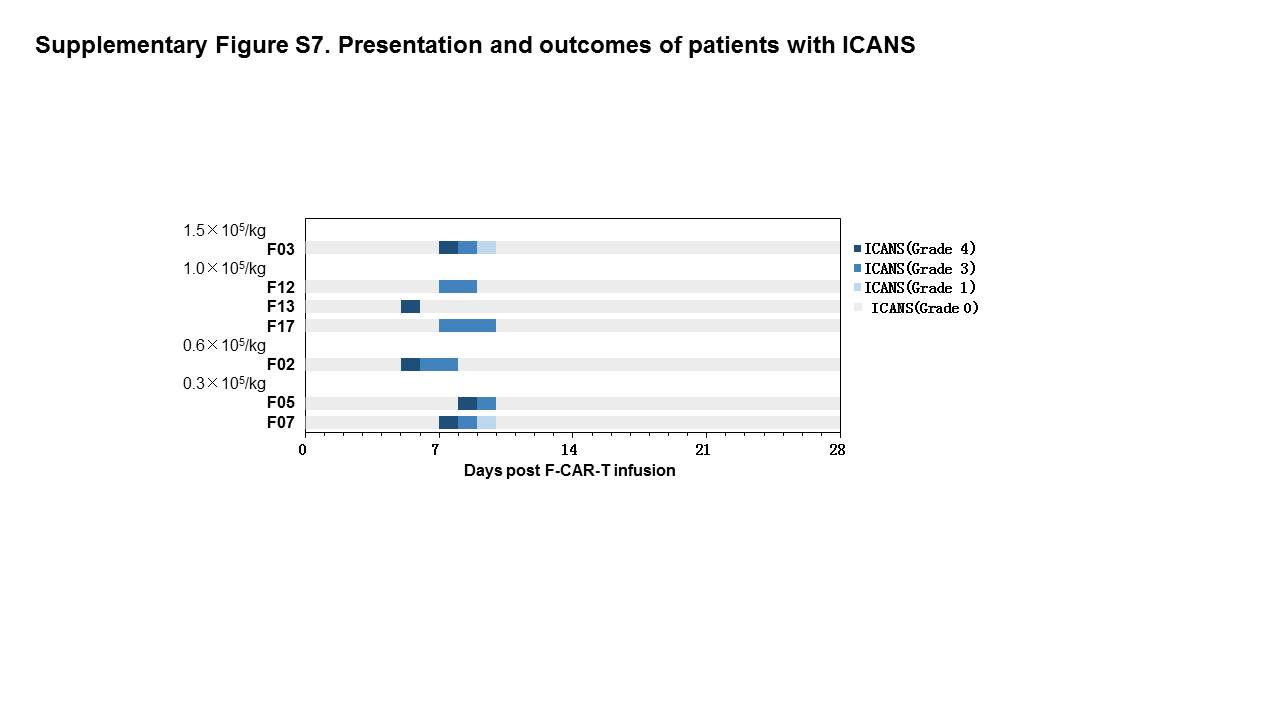
**

**Supplementary Figure S7. Presentation and outcomes of patients with ICANS**

ICANS was observed in 7(28%) patients, with 2(8%) grade 3, 5(20%) grade 4, and no grade 5 ICANS developed. The symptoms of neurotoxicity occurred within 14 days post infusion with a median duration of 3 days(range, 1-3).

ICANS: immune effector cell-associated neurotoxicity syndrome.

**
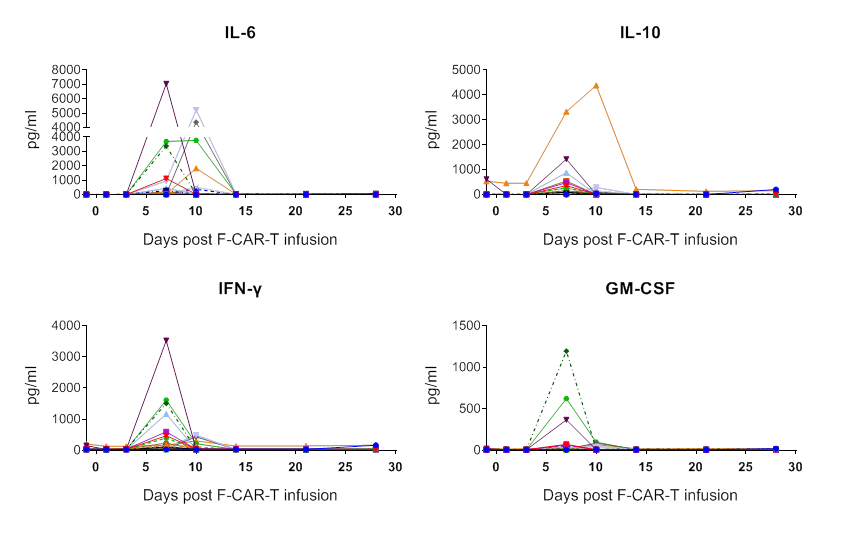

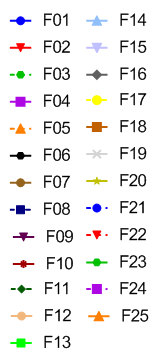
**

**Supplementary Figure S8. Plasma cytokine levels after F-CAR-T infusion**

The change of IL-6, IFN-γ, IL-10, and GM-CSF level post F-CAR-T infusion were selectively shown. The peak levels of those 4 cytokines were observed around day 7-10.


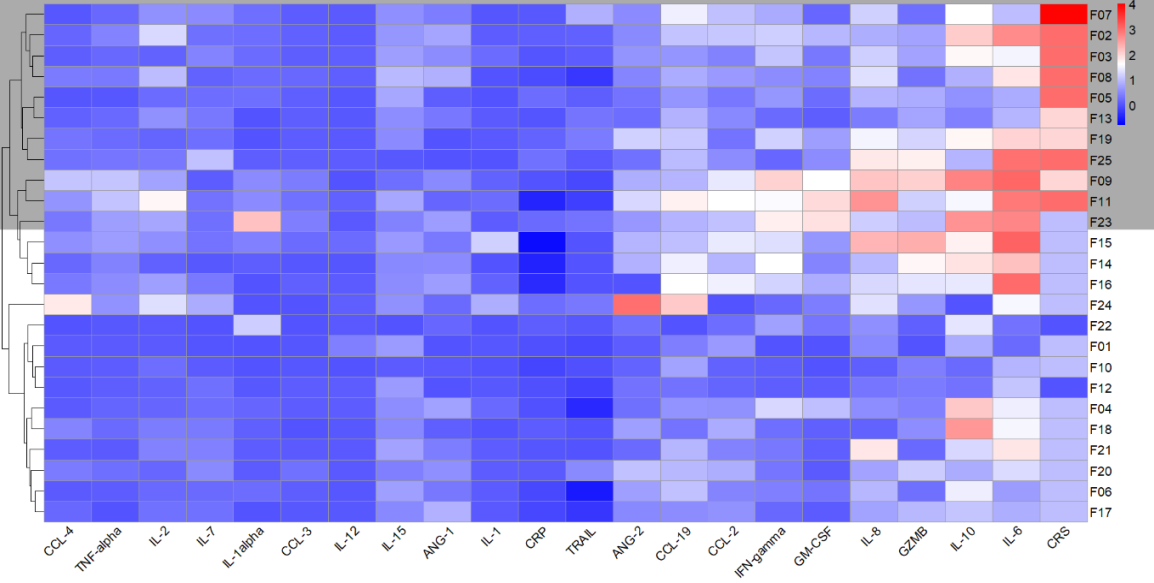

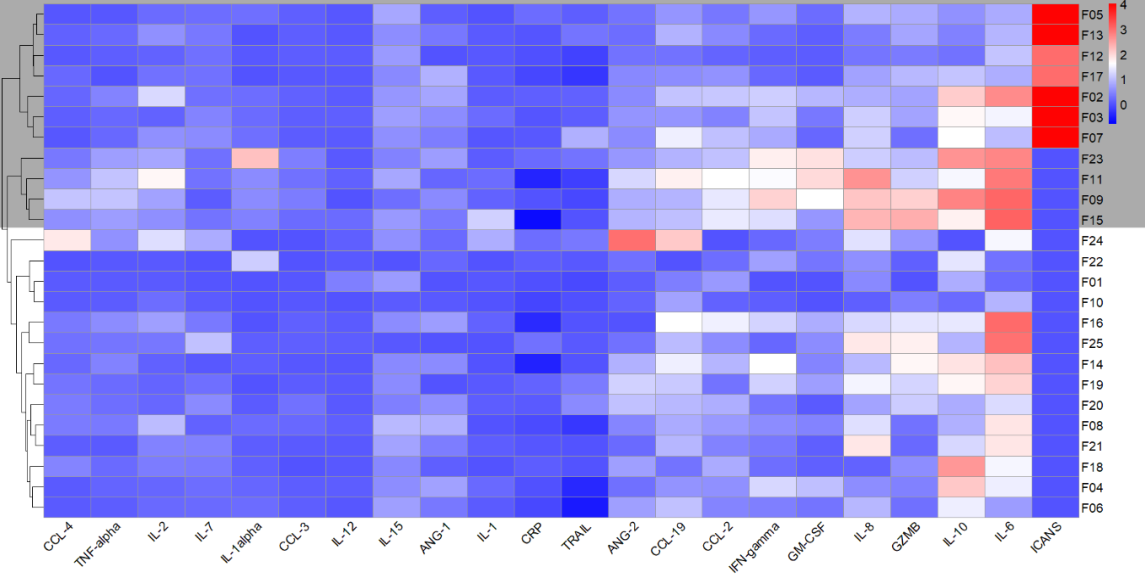


**Supplementary Figure S9. Association of cytokines post infusion with severe CRS and ICANS**

Among all 21 cytokines examined, only IL-6 level post infusion was associated with moderate to severe CRS and ICANS.

CRS: Cytokine release syndrome; ICANS: immune effector cell-associated neurotoxicity syndrome.

**Supplementary Figure S10. Association of cytokines before infusion with severe CRS and ICANS**


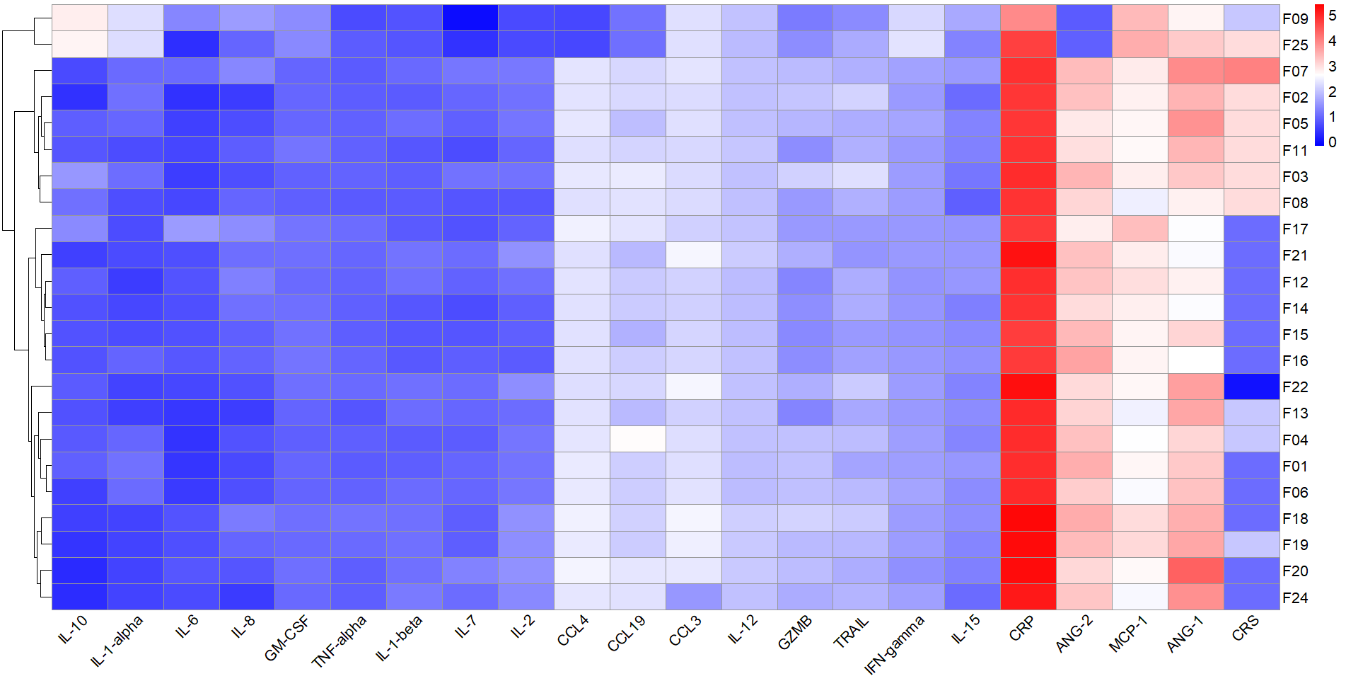

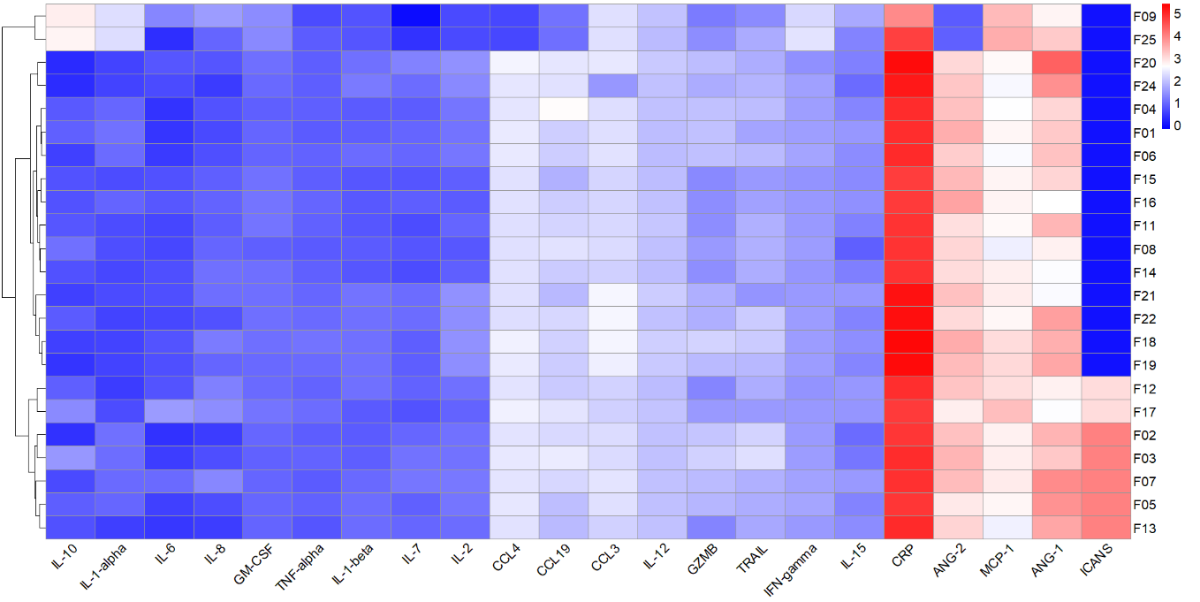


CRS: Cytokine release syndrome; ICANS: immune effector cell-associated neurotoxicity syndrome.


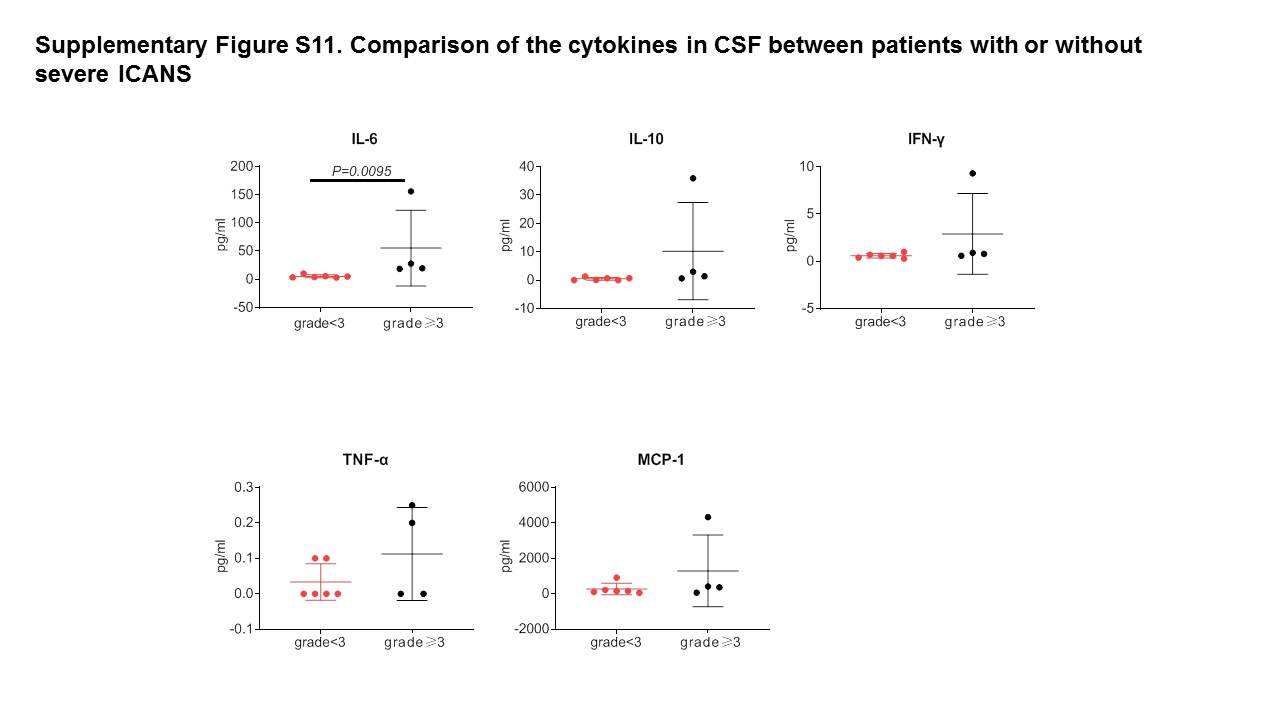


**Supplementary Figure S11. Comparison of the cytokines in CSF between patients with or without severe ICANS**

Patients with severe ICANS had higher IL-6 level in contrast to patients without severe ICANS, and the difference between the median level of IL-6 among these two groups of patients was statistically significant, while no significant difference was observed in other 4 cytokines between the two groups of patients. Statistical significance was determined by Mann-Whitney test.

CSF: cerebrospinal fluid; ICANS: immune effector cell-associated neurotoxicity syndrome.

## Supplementary Tables

**Table S1. FasT CAR-T release test and criteria**

| Sampling stage | Category | Parameter | Method | Criteria |
| --- | --- | --- | --- | --- |
| At end of culture | Safety | Copies number of CAR | qPCR | ≤5.00 copies/cell |
|  |  | Mycoplasma | qPCR (quick test) | Undetectable |
|  |  | Mycoplasma | Pharmacopoeia method | Undetectable |
|  | Identity | CAR gene sequence | DNA sequencing | 100% matching |
|  |  | Percentage of T cells | Flow cytometry | ≥80.0% |
|  |  | Residual CD19-positive cells | Flow cytometry | ≤1.0% |
| After filling | Safety | Endotoxin | Gel Clot LAL Assay | ≤3EU/mL |
|  |  | Sterility | Blood culture (quick test) | Undetectable |
|  |  | Sterility | Pharmacopoeia method | Undetectable |
| Product | Safety | Appearance | Visual inspection | Colorless to light yellow cell suspension |
|  | Potency | Viability | AO/DAPI staining | ≥70.0% |
| 72h after thawing | Content | CAR-T cell number | AO/DAPI staining and cell counting | ≥dosage×1.1 |
|  | Potency | CAR positive rate | Flow cytometry | ≥10.0% |
|  |  | IFN-γ | ELISA | ≥2.00ng/mL |

Abbreviations: AO: acridine orange; DAPI: 4',6-diamidino-2-phenylindole.

**Table S2. Gene sets**

| Cell cycle | Apoptosis |
| --- | --- |
| ADD1 | AKT1 |
| AKT1 | AKT2 |
| AKT2 | AURKA |
| APC | BUB1 |
| ATG14 | CD7 |
| BATF3 | CDC26 |
| BCL2 | CDKN1A |
| BID | CKAP5 |
| CASP3 | GADD45B |
| CASP8 | JAK2 |
| CD14 | MAX |
| CTNNA1 | MX1 |
| CTNNB1 | NEK2 |
| CTSD | NSD2 |
| CTSW | PPP2R5D |
| DIABLO | PRKCB |
| FAM30A | RAC2 |
| FAS | RBX1 |
| FOS | SGK3 |
| GADD45B | SKP1 |
| GZMA | SLC7A5 |
| GZMB | SMAD2 |
| GZMH | SMARCA4 |
| GZMM | TCL1A |
| IL3 | TFDP1 |
| IL37 | TGFB1 |
| JUN | TP53 |
| MAGED1 | TPR |
| MAP2K2 | UBE2I |
| MAP3K14 |  |
| MAPK3 |  |
| MKI67 |  |
| NCAPD2 |  |
| NCAPG2 |  |
| NCAPH |  |
| NFKBIA |  |
| NMT1 |  |
| OMA1 |  |
| OPA1 |  |
| PARP1 |  |
| PIDD1 |  |
| PIK3R1 |  |
| PIK3R2 |  |
| PIK3R3 |  |
| PRF1 |  |
| PRKCD |  |
| PSMA2 |  |
| PSMA3 |  |
| PSMA6 |  |
| PSMB10 |  |
| RELA |  |
| STAT3 |  |
| TFDP1 |  |
| TICAM1 |  |
| TLR4 |  |
| TNF |  |
| TNFRSF10B |  |
| TP53 |  |

**Table S3. Bridging chemotherapy prior to FasT CAR-T cells**

| **Patient No.** | **Bridging Chemotherapy Regimen** |
| --- | --- |
| F01 | / |
| F02 | VLD |
| F03 | L-asp+DEX |
| F04 | MTX+Ara-c+6-MP+L-asp |
| F05 | MTX |
| F06 | MTX |
| F07 | VLD |
| F08 | MTX |
| F09 | VLD+Chidamide+Sirolimus |
| F10 | VLD |
| F11 | VLD |
| F12 | Ara-c+6-MP+L-asp |
| F13 | VLD |
| F14 | VLD |
| F15 | VLD |
| F16 | Bortezomib+L-asp+VDS |
| F17 | MTX+DEX |
| F18 | VLD |
| F19 | VLD+IDA |
| F20 | IDA |
| F21 | Flu+Ara-c |
| F22 | / |
| F23 | VLD+6-MP |
| F24 | / |
| F25 | Ara-c+6-MP+L-asp |

Abbreviations:

VDS: vindesine; L-asp: L-asparaginase; DEX: dexamethasone; MTX: methotrexate;

Ara-c: cytarabine; 6-MP: mercaptopurine; VLD: vincristine, L-asparaginase, dexamethasone; VDLP: vincristine, daunorubicin, asparaginase, prednisone; IDA: idarubicin; Flu: fludarabine

**Table S4. CRS and ICANS are grouped by age**

| Variable |  | | All patients  (N=25) | | <14yr  (N=5) | ≥14yr  (N=20) | | |
| --- | --- | --- | --- | --- | --- | --- | --- | --- |
|  |  | | *number of patients (percent)* | | | | | |
| CRS | Any grade | | 24(6) | | 5(100) | | | 19(95) |
|  | Grade 1 | | 14(56) | | 0 | | | 14(70) |
|  | Grade 2 | | 4(16) | | 2(40) | | | 2(10) |
|  | Grade 3 | | 6(24) | | 3(60) | | | 3 (15) |
| ICANS | Any grade | 7(28) | | 5(100) | | | 2(10) | |
|  | Grade 1-2 | 0 | | 0 | | | 0 | |
|  | Grade 3 | 2(8) | | 0 | | | 2(10) | |
|  | Grade 4 | 5(20) | | 5(100) | | | 0 | |

Abbreviations: CRS, cytokine release syndrome; ICANS, immune effector cell-associated neurotoxicity syndrome; yr, years.

**Table S5. Clinical correlations with cytokines of CSF and PB**

| **Pt. No** | **Test**  **date** | **CAR-T/T %** | **CAR-T cells/ml** | **IL-1b (pg/ml)** | **IL-6 (pg/ml)** | **IL-10 (pg/ml)** | **IFN-γ**  **(pg/ml)** | **TNF-α**  **(pg/ml)** | **MCP-1 (pg/ml)** | **GM-CSF (pg/ml)** | **CAR-T cell/kg** | **CRS** | **ICANS** |
| --- | --- | --- | --- | --- | --- | --- | --- | --- | --- | --- | --- | --- | --- |
|  |  | **CSF/PB** | **CSF/PB** | **CSF/PB** | **CSF/PB** | **CSF/PB** | **CSF/PB** | **CSF/PB** | **CSF/PB** | **CSF/PB** |  |  |  |
| F03 | D7 | /0.8 | /629 | / | / | / | / | / | / | / | 1.47×10^5^ | 3 | 4 |
|  | D10 | 77.4/50.4 | /245000 | 0/ | 28.1/ | 3.0/ | 0.9/ | 0/ | 62.8/ | 0/ |  |  |  |
| F04 | D7 | /25.4 | /1710 | /7.1 | /60.0 | /480.0 | /599.4 | /8.8 | /146.6 | /66.8 | 1.56×10^5^ | 2 | 0 |
|  | D10 | /34.6 | /813000 | /6.9 | /1.9 | /6.9 | /37.3 | /6.0 | /67.7 | /7.6 |  |  |  |
|  | D16 | 79.2/9.5 | 3969/56100 | 0/10.2 | 5.8/4.0 | 0.7/8.5 | 0.6/43.5 | 0/7.5 | 54.4/112.9 | 0/8.0 |  |  |  |
| F06 | D7 | /69.4 | /7860 | /10.7 | /13.6 | /97.7 | /94.0 | /9.3 | /325.2 | /15.5 | 6.62×10^4^ | 1 | 0 |
|  | D10 | /58.2 | /92700 | /9.3 | /3.7 | /4.4 | /40.9 | /7.7 | /85.4 | /8.3 |  |  |  |
|  | D15 | 73.9/6.6 | 5760/4060 | 0/9.7 | 3.2/4.0 | 0.4/4.8 | 0.6/40.1 | 0.1/7.2 | 101.4/79.5 | 0/9.2 |  |  |  |
|  | D101 | 2.4/0 | 25/ NA | 0/ NA | 7.2/ NA | 0.7/ NA | 1.7 /NA | 0/ NA | 196.9/ NA | NA |  |  |  |
| F07 | D7 | /0.1 | /15 | / | / | / | / | / | / | / | 3.0×10^4^ | 4 | 4 |
|  | D9 | 43.7/9.6 | /4060 | 0/ | 155.9/ | 35.9/ | 9.3/ | 0/ | 4323.4/ | 0/ |  |  |  |
| F08 | D7 | /3.3 | /2040 | /6.4 | /283.3 | /81.5 | /110.6 | /13.9 | /1192.0 | /19.9 | 7.04×10^4^ | 3 | 0 |
|  | D10 | /63 | /119000 | /5.4 | /9.6 | /6.0 | /30.9 | /5.6 | /45.2 | /6.7 |  |  |  |
|  | D20 | 63.3/6.5 | 815/2840 | 0/5.1 | 5/2.6 | 1.3/4.9 | 0.4/28.7 | 0/5.8 | 219.2/126.9 | 0.3/7.5 |  |  |  |
| F10 | D7 | /20.1 | /244 | /6.3 | /17.2 | /7.3 | /32.8 | /5.7 | /374.2 | /6.3 | 5.86×10^4^ | 1 | 0 |
|  | D10 | /22.4 | /12700 | /6.7 | /1.8 | /4.4 | /23.9 | /4.1 | /109.3 | /5.7 |  |  |  |
|  | D17 | 2.17/8.3 | 97/4980 | 0/7.4 | 3.6/2.1 | 0/4.8 | 0.7/26.2 | 0/4.6 | 151.5/106.1 | 0/6.3 |  |  |  |
| F12 | D7 | /57 | /22100 | /1.5 | /0.7 | /0 | /3.2 | /2.1 | /19.8 | /1.0 | 6.50×10^4^ | 1 | 3 |
|  | D10 | /42.9 | /34500 | /9.2 | /41.4 | /7.5 | /27.9 | /8.6 | /213.5 | /9.0 |  |  |  |
|  | D14 | 40.6/11.9 | 2696/NA | 0/10.4 | 19.6/12.0 | 1.4/7.1 | 0.8/26.7 | 0.2/6.9 | 411/103.1 | 0/9.5 |  |  |  |
| F13 | D7 | /61.9 | /10000 | / | / | / | / | / | / | / | 1.02×10^5^ | 2 | 4 |
|  | D10 | /8.3 | /26800 | / | / | / | / | / | / | / |  |  |  |
|  | D14 | 61.1/2.1 | /6940 | 0/ | 18.6/ | 0.6/ | 0.6/ | 0.3/ | 360.9/ | 0/ |  |  |  |
| F14 | D7 | /59.1 | /1060 | /6.3 | /435.0 | /875.4 | /1169.0 | /15.5 | /1230.0 | /32.7 | 6.35×10^4^ | 1 | 0 |
|  | D10 | /56.8 | /189000 | /5.6 | /40.7 | /5.1 | /33.1 | /8.2 | /122.1 | /11.4 |  |  |  |
|  | D31 | 50.4/0.1 | 1128/400 | 0/5.3 | 4.1/2.7 | 0/5.2 | 1/23.4 | 0/6.5 | 157.3/67.7 | 0/12.2 |  |  |  |
| F15 | D7 | /0 | /306 | /6.9 | /940.3 | /35.7 | /95.2 | /19.6 | /1578.0 | /32.7 | 6.0×10^4^ | 1 | 0 |
|  | D10 | /35.9 | /23500 | /91.0 | /5219.0 | /285.3 | /495.3 | /33.5 | /7563.0 | /47.9 |  |  |  |
|  | D17 | 1.3/0.3 | 12/7650 | 0/6.3 | 10.3/43.6 | 0.1/5.4 | 0.3/28.2 | 0.1/8.2 | 912.8/226.8 | 0/15 |  |  |  |
|  | D20 | /2.6 | /NA | /5.6 | /26.9 | /4.9 | /26.6 | /6.1 | /242.9 | /11.4 |  |  |  |
| F16 | D7 | /2.7 | /25 | /6.3 | /25.1 | /8.8 | /43.7 | /9.5 | /1537.0 | /15.0 | 0.99×10^5^ | 1 | 0 |
|  | D10 | /6.7 | /4400 | /9.4 | /4376.0 | /93.5 | /439.3 | /31.6 | /13156.0 | /89.5 |  |  |  |
|  | D14 | /10.3 | /131000 | /5.6 | /17.3 | /4.0 | /33.1 | /37.8 | /157.3 | /11.4 |  |  |  |
|  | D29 | 3.57/0 | 89/707 | NA/5.6 | NA/26.5 | NA/3.8 | NA/29.8 | NA/10.8 | NA/308.9 | NA/13.1 |  |  |  |
| F20 | D7 | /65.7 | /222000 | /10.9 | /63.6 | /13.7 | /40.4 | /7.8 | /167.7 | /8.0 | 1.03×10^5^ | 1 | 0 |
|  | D10 | /19.1 | /288000 | /11.9 | /20.7 | /4.7 | /27.8 | /7.8 | /137.6 | /10.6 |  |  |  |
|  | D15 | 60.4/4.6 | 1053/15800 | NA/9.8 | NA/26.9 | NA/6.6 | NA/30.8 | NA/8.6 | NA/150.1 | NA/10.6 |  |  |  |
| F24 | D7 | /14.5 | /41700 | /13.0 | /43.6 | /28.6 | /121.9 | /14.8 | /396.2 | /53.9 | 6×10^4^ | 1 | 0 |
|  | D10 | /18.6 | /122000 | /14.2 | /3.4 | /1.9 | /33.9 | /7.8 | /48.0 | /6.8 |  |  |  |
|  | D32 | 0.5/0.2 | 2/8000 | NA/11.9 | NA/3.8 | NA/2.4 | NA/37.1 | NA/7.8 | NA/103.1 | NA/6.8 |  |  |  |

Abbreviations: pt, patient; BM: bone marrow; PB, peripheral blood; CRS, cytokine release syndrome; ICANS, immune effector cell-associated neurotoxicity syndrome; MRD, minimal residual disease; CR, complete remission; CRi, CR with incomplete blood count recovery. NA, not available.

**Table S6. Flow antibody list**

| Reagent | Company, & Cat# |
| --- | --- |
| FITC-anti-CD4 | BD, Cat#340133 |
| APC-H7- anti-CD8 | BD, Cat#560179 |
| BV510- anti-CD45RA | BD, Cat#563031 |
| PE- anti-CCR7 | Invitrogen, Cat#12-1979-42 |
| BV421 anti-CD62L | BD,Cat#563862 |
| APC-anti-CD45RO | BD, Cat#340438 |
| FITC-anti-CD3 | ebioscience, Cat #340542 |
| APC-anti-LAG-3 | Ebioscience, Cat# 17-2239-42 |
| BV421-antiPD-1 | Biolegend, Cat#329920 |
| PE-Cy7-antiTim3 | Biolegend, Cat# 345014 |
| PE-anti-DYKDDDDK | Biolegend, Cat#637310 |
| BD Horizon™ BV510 Mouse Anti-Human CD45 | BD, Cat#563204 |
| [BD Pharmingen™ PE-Cy™7 Mouse Anti-Human CD4](https://www.bdbiosciences.com/cn/applications/research/t-cell-immunology/th-1-cells/surface-markers/human/pe-cy7-mouse-anti-human-cd4-sk3-also-known-as-leu3a/p/557852) | BD, Cat#557852 |
| BD Pharmingen™ PE Mouse Anti-Human CD2 | [BD, Cat#555327](https://www.bdbiosciences.com/cn/applications/research/t-cell-immunology/regulatory-t-cells/surface-markers/human/pe-mouse-anti-human-cd2-rpa-210/p/555327) |
| Biotin rabbit anti-human FMC63 scfv | BIOSWAN, R19PB100 |
| Streptavidin APC | Invitrogen, SA1005 |
| PE/Cy7 anti-human CD184 (CXCR4) Antibody | BD, Cat#306514 |
| 7-AAD solution | BD, Cat#51-68981E |
| Lysing buffer(10X) | BD, Cat#555899 |
